# Integrated serum pharmacochemistry, 16S rDNA sequencing, and metabolomics to reveal the material basis and mechanism of Shouhui Tongbian capsule against diphenoxylate-induced slow transit constipation in rats

**DOI:** 10.1186/s13020-024-01015-8

**Published:** 2024-10-11

**Authors:** Jiaying Yang, He Xiao, Jingchun Yao, Pin Zhang, Bojiao Yi, Zhengyu Fang, Na Guo, Yongxia Guan, Guimin Zhang

**Affiliations:** 1 Experimental Research Center, China Academy of Chinese Medical Sciences, Beijing, 100700 China; 2 College of Pharmacy, Heilongjiang University of Traditional Chinese Medicine, Harbin, 150040 Heilongjiang China; 3 State Key Laboratory of Integration and Innovation of Classic Formula and Modern Chinese Medicine, Lunan Pharmaceutical Group Co. Ltd, Shandong, 273400 Linyi China; 4 School of Pharmacy, Shenyang Pharmaceutical University, Shenyang, 110016 Liaoning China

**Keywords:** Slow transit constipation, Chinese medicine, Lipidomics, Intestinal microflora, Lipidomics, Short-chain fatty acid

## Abstract

**Background:**

Slow transit constipation (STC) is highly prevalent and has rising incidence. Shouhui Tongbian capsule (SHTB) is a traditional Chinese Medicine formula with extensive and highly efficacious usage in STC treatment, however, its mechanism of action, especially the regulation of microbiome and lipid metabolites, remains unclear.

**Methods:**

After quality control of SHTB using LC‒MS to obtain its material basis, we tried to elucidate the cohesive modulatory network of SHTB against STC using hyphenated methods from microbiomics, lipidomics, mass spectrometry imaging (MSI) and molecular methods.

**Results:**

SHTB could repair intestinal barrier damage, reduce systemic inflammation and increase intestinal motility in a diphenoxylate-induced STC rat model. Based on 16S rDNA sequencing results, SHTB rehabilitated the abnormal changes in *Alloprevotella, Coprococcus, Marvinbryantia,* etc., which were associated with STC symptoms. Meanwhile, microbial functional prediction showed that lipid metabolism was improved with SHTB administration. The differential lipids, including fatty acids, lysophosphatidylcholine, phosphatidylcholine, sphingomyelin triglyceride and ceramide, that are closely related to STC disease and SHTB efficacy. Furthermore, SHTB significantly reversed the abnormal expression of these key target enzymes in colon samples, including CTP-phosphocholine cytidylyltransferase, CTP-phosphoethanolamine cytidylyltransferase, phosphatidic acid phosphatase, acid sphingomyelinase etc.

**Conclusions:**

Combined analysis demonstrated that SHTB reducing lipid accumulation and recovery of intestinal microbial homeostasis was the critical mechanism by which SHTB treats STC.

**Supplementary Information:**

The online version contains supplementary material available at 10.1186/s13020-024-01015-8.

## Introduction

Constipation is a highly prevalent gastrointestinal tract disorder affecting 5–20% of the global population [1, 2], causing poor quality of life and high healthcare costs [1]. Based on sites of attack and characteristics of colonic motility, constipation can be separated into three types: mixed, outlet obstruction, and slow transit constipation (STC) [3]. STC is the most frequently occurring form of functional constipation. It severely impacts the physical and mental condition of patients and enhances financial burden on the healthcare system [4]. More recently, traditional Chinese medicines (TCMs) are increasingly used in STC therapy owing to its increased efficacy, reduced toxicity and cost effectiveness [5]. Shouhui Tongbian Capsule (SHTB), composed of eight herbs (i.e., Atractylodes macrocephala, Ginseng, *Angelica sinensis*, *Polygonum multiflorum*, *Aloe vera*, *Semen Cassiae*, Wolfberry, and Aurantii Fructus Immaturus), is applied in the treatment of gastrointestinal motility disorders clinically and has good effects on STC [6]. In clinical applications, SHTB effectively reduced the symptoms and physical signs of STC patients and improved the anorectal dynamics index as well as the quality of life of patients [7]. However, systematic reports are limited on the quality control, efficacy, and mechanism of SHTB, especially from the perspectives of gut microbiota and lipid metabolism homeostasis.

Recently, increasing evidence has indicated dysregulated gut microbiota is a likely risk factor for constipation [8]. For instance, emerging evidences [9] reveal that intestinal dysbiosis causes constipation. Antibiotic-administered mice who received fecal microbiota from constipated individuals exhibited unusual defecation. Notably, inflammatory responses and impaired colonic epithelial integrity potentially cause this disorder of gut microbiota, resulting in reduced bowel discharge. The latter disrupts colonic sensorimotor abilities and brings about abnormalities in neurotransmitters and neurochemical signals, thus reducing intestinal peristalsis and promoting constipation [10, 11]. Additionally, increased fat intake could induce intestinal oxidative stress, directly affecting intestinal barrier function and activating inflammatory reactions, hence causing an imbalance in gut microbiota composition [12]. Therefore, it is promising to elucidate the underlying mechanism of SHTB by investigating the composition and physiological roles of gut microbiota regulated by SHTB. The introduction of high-throughput sequencing, namely 16S rDNA and metabolomics, has paved way for culture-independent methods of overall gut microbiota examination, instead of exploration of individual microbes. This facilitated the elucidation of the functions of certain microbes and microbial mediators that regulate constipation [13]. Prior investigations primarily focused on the association between alternating intestinal flora and disease states [14, 15]; in contrast, emerging studies have defined mechanisms of gut microbiota that contribute to constipation-associated symptoms [15]. Nonetheless, the underlying mechanisms of gut microbiota–host associations by SHTB regulation remain to be elucidated, particularly those modulating the lipid metabolism system.

Herein, we verified the mitigating impacts of SHTB on STC and to investigate whether the mechanism is through the mediation of gut microbiota and lipid metabolism. We first conducted quality control and blood component analysis of SHTB according to liquid chromatography‒mass spectrometry (LC‒MS) technology to unravel the material basis behind SHTB. Using a diphenoxylate-induced STC rat model, we then evaluated the ameliorative influences of SHTB on gut microbiota dysbiosis, intestinal barrier damage, and inflammatory responses caused by STC based on 16S rDNA sequencing technology and ELISA detection. Thereafter, targeted metabolomics analyses and MSI technology were used to assess changes in lipid metabolites in the serum, feces, liver, and intestine tissues after SHTB treatment. To verify the regulatory role of SHTB on key enzymes of lipid metabolism and better understand the underlying signaling governing SHTB-mediated STC intervention.

## Materials and methods

### Chemicals

SHTB, Maren pills (MRW), and diphenoxylate were acquired from Lunan Pharmaceutical Group Corporation (Shandong, China); reference standards of SHTB from Beijing Rongchengxinde Technology Co., Ltd, Push Bio-Technology, Shanghai Yuanye Bio-Technology Co., Ltd, Chengdu Alfa Biotechnology, Guangzhou Juntang Technology Co., Ltd. and Institute of Medicinal Plant Development (Chinese Academy of Medical Sciences) in China; standards for rat endogenous compounds from Sigma‒Aldrich (MO, USA), Cambridge Isotope Laboratories (Andover, MA, United States), Tokyo Chemical Industry (Tokyo, Japan), Toronto Research Chemicals (YTO, Canada), Larodan (Stockholm, Sweden), and Avanti Polar Lipids (Alabama, USA); LPS and diamine oxidase (DAO) assay kit from Nanjing Jiancheng Bioengineering Institute (Nanjing, China) and Xiamen Limulus Reagent Experimental Factory (Xiamen, China), respectively; tumor necrosis factor-alpha (TNF-α) and interleukin 1β (IL-1β) assay kits from Beijing North Institute of Biological Engineering (Beijing, China); the phosphatidic acid phosphatase (PAP), acid sphingomyelinase (ASMase), sphingomyelin synthase (SMS), adipose triglyceride lipase (ATGL) and hormone-sensitive lipase (HSL) ELISA kits from COIBO BIO (Shanghai, China); PCYT1A antibody (Bioss/bs-11306) and PCYT2 antibody (Bioss/bs-11663) from Bioss Antibodies Company (Beijing, China); acetonitrile (LC‒MS grade) and methanol (LC‒MS grade) from Fisher Scientific; formic acid (LC‒MS grade) from Sigma‒Aldrich; concentrated sulfuric acid, hydrochloric acid, and phosphoric acid of analytical grade from China National Pharmaceutical Group Corporation; and a water purification system (Milli-Q; Millipore, Bedford, MA, United States of America) was employed for ultrapure water (18.2 MΩ) preparation.

### Analysis of the chemical constituents of SHTB via UPLC-Q-TOF–MS

Each SHTB batch was dissolved in 60% ethanol to a total volume of 20 mL. The solutions were further diluted 10 and 100 times for systematic screening of chemical compositions and quantification of main ingredients in SHTB, respectively.

**Fig. 1:**
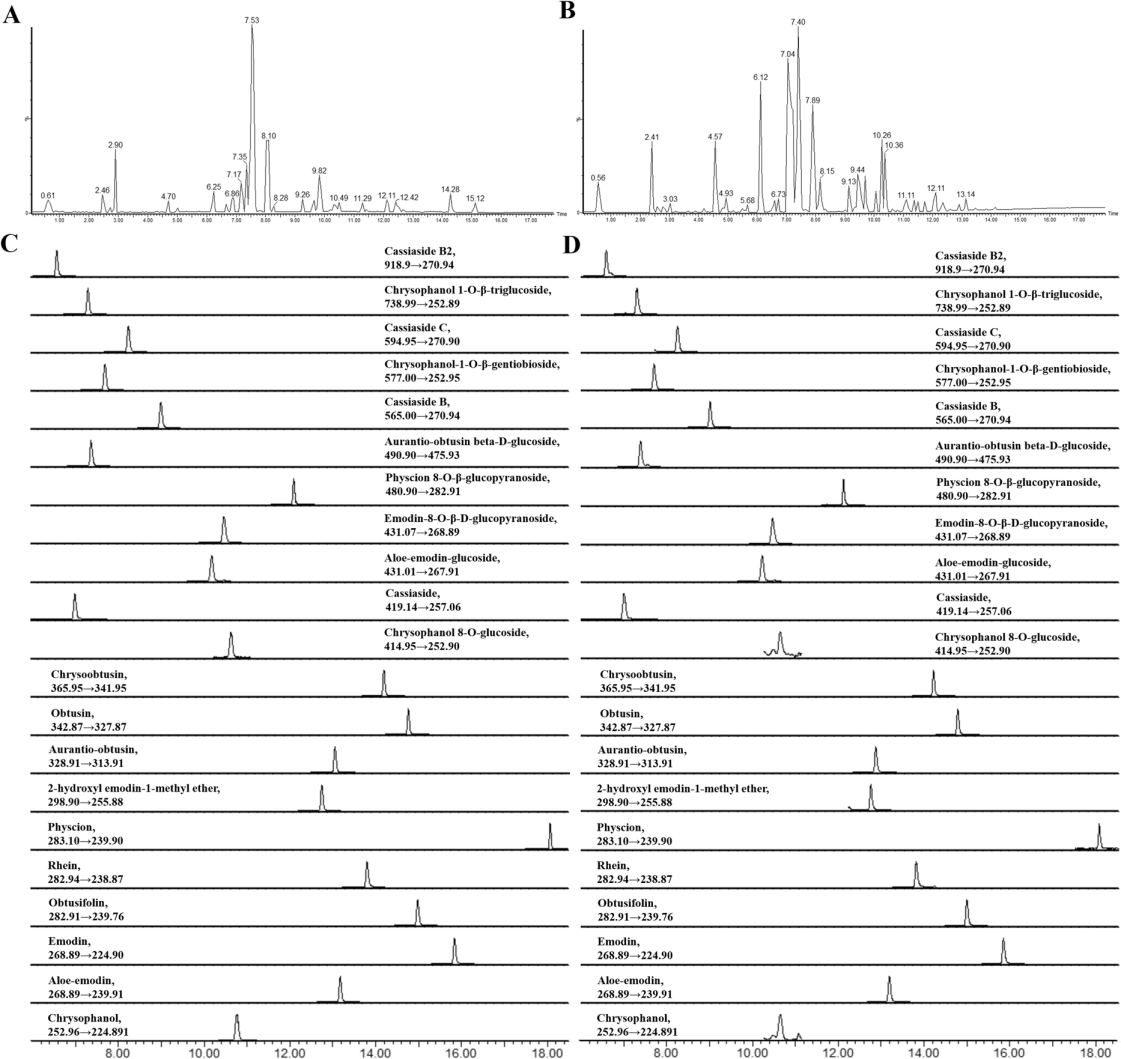
Qualitative and quantitative analysis of the chemical compounds in SHTB capsules. Typical BPI chromatogram for SHTB samples obtained in **A** ESI + mode and **B** ESI- mode based on UPLC-Q-TOF–MS. **C** Typical UPLC‒MS/MS chromatograms of the 21 compounds in mixed standard solution. **D** Typical UPLC‒MS/MS chromatograms of the 21 compounds in the SHTB capsule

Systematic screening of chemical constituents was conducted using a Waters ACQUITY UPLC system attached to a Waters Xevo G2XS Q-TOF system (Waters, Milford, MA, USA) with an electrospray ionization (ESI) source in positive and negative ion modes. The chromatographic separation and mass spectrometric analysis procedures are described in Supplementary Note 1.1. Typical BPI chromatograms for SHTB samples in ESI + and ESI- modes are depicted in Fig. 1A, B.

We revealed that the anthraquinones and naphthopyranones are important components of *Polygonum multiflorum*, aloe vera, and *Cassia seed* compounds with laxative, antibacterial, and anti-inflammatory effects [16]. Subsequently, 21 components of anthraquinones and naphthopyranones were selected for further quantification via the Waters ACQUITY UPLC system attached to a Waters Xevo TQ-S microsystem (Waters, Milford, MA, USA) in ESI negative ion mode. The chromatographic conditions were similar to those used in the systematic screening (Supplementary Note 1.1), while the mass spectrometry parameters are described in Supplementary Note 1.2. Typical UPLC‒MS/MS chromatograms of the 21 compounds in combined standard reagents and SHTB capsules (Fig. 1). The TargetLynx program (v 4.1, Waters, Milford, MA) was employed for primary ingredient quantification before validating the corresponding method (Supplementary Note 1.2).

### Animal treatments

**Fig. 2:**
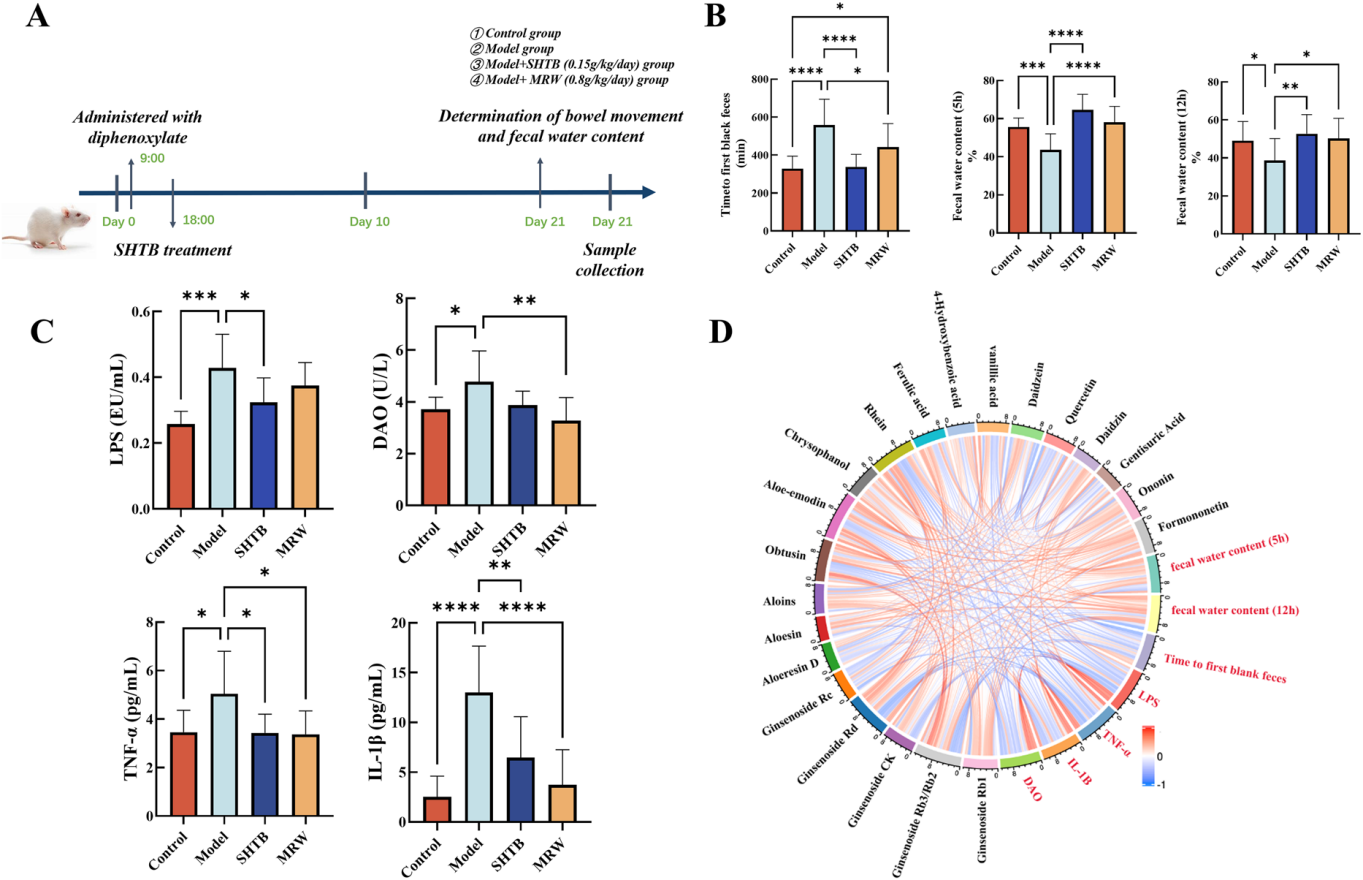
SHTB alleviated STC symptoms induced in response to diphenoxylate in rats. **A** Study flow chart. **B** Effects of SHTB on the fecal parameters in rats. **C** The serum levels of lipopolysaccharide (LPS), diamine oxidase (DAO), tumor necrosis factor-alpha (TNF-α), and interleukin 1β (IL-1β) were detected by ELISA kits. **D** Correlation analysis between serum prototype components of SHTB and constipation-related parameters. Data are means ± SDs and were compared using Student’s t tests. n = 8–14 per group. Control is the control group; Model is the constipation model group; SHTB is the treatment group with the SHTB group; MRW is the treatment group with the MRW group. *p < 0.05; **p < 0.01; ***p < 0.001; ****p < 0.0001

All animal protocols received ethical approval from the Institutional Animal Care and Use Committee (IACUC) of Lunan Pharmaceutical Group Corporation (AN-IAUUC-2021-046). Sixty healthy female specific pathogen-free (SPF) rats (average weight 200 ± 30 g) were acquired from the same group and kept in an animal laboratory (relative humidity 60% ± 5%, 12 h/12 h light/dark cycle at 22 ± 2 °C, with freely available water and food). Following 1-week of acclimatization, they were randomized into four cohorts (n = 15 per cohort): control, model, SHTB (0.15 g/kg), and a positive control group MRW (0.8 g/kg). Figure 2A shows the study flow chart. For the model group, we administered a water suspension of diphenoxylate (10 mg/kg) at 09:00 and 18:00. Control rats received normal saline (10 mL/kg) at both time points. Rats in the SHTB and MRW groups also received a water suspension of diphenoxylate (10 mg/kg) at 09:00, followed by SHTB (0.15 g/kg) and MRW (0.8 g/kg) at 18:00. All rats received daily intragastric administration for 20 days.

### Determination of bowel movement and fecal water content

After treatments, rats were only allowed water for the next 16 h. Thereafter, the control group and the other three groups were treated with normal saline and a water suspension of diphenoxylate (10 mg/kg) via oral administration. After 30 min, the control and model groups were orally administered charcoal meal (3% activated charcoal suspended in 0.5% aqueous methylcellulose; Sigma Aldrich). The SHTB and MRW groups were given charcoal meal containing SHTB (0.15 g/kg) and MRW (0.8 g/kg), respectively. The gastric perfusion volume was 20 mL/kg. Then, individual rats were placed in a distinct metal cage with freely available food and water.

Bowel movement per rat was assessed based on the duration of the charcoal meal provision to the first defecation of black feces. The weight and number of all black feces between 5 and 12 h were recorded for each rat. The water content of feces between 5 and 12 h was analyzed as shown below:

fecal water content (%) = (A–B)/A × 100%, whereby A denotes fresh fecal pellet weight, and B denotes dried (at 60 °C for 5 h) fecal pellet weight.

### Sample collection

Abdominal massage was performed on the rats to stimulate defecation. After discarding the first stool, subsequent fecal samples of each rat were collected into aseptic freezing tubes, and flash frozen in liquid nitrogen prior to storage at – 80 °C until additional analyses. Subsequently, anesthesia was provided via a 30-min intraperitoneal sodium pentobarbital (50 mg/kg) perfusion. Blood was drawn from the rats via the jugular veins prior to a 10-min centrifugation at 3000 g at 4 °C for serum separation, which was maintained at − 80 °C until further analyses.

### Assessment of the active serum prototype components of SHTB

A Waters Xevo G2XS Q-TOF system (Waters Corp., Milford, MA, USA) attached to an electrospray ionization (ESI) source was used for qualitative analyses of the active serum prototype components of SHTB in both positive and negative ion modes. The instrument calibration method, LC, and MS protocols was the same as in "Chemicals" section. .

### 16S rDNA sequencing of fecal samples

Following collection as directed in "Sample collection" section, fecal samples were treated with the FastDNA^®^ Spin Kit for Soil (MP Biomedicals, USA) for total DNA extraction. Using a Nanodrop 2000 UV‒Vis Spectrophotometer (NanoDrop Technologies, Wilmington, USA), we measured the quality and quantity of isolated DNA samples, whereas their integrity was determined using a 1% agarose gel (5 V/cm, 20 min). The V3-V4 region of 16S rDNA was PCR-amplified using forward and reverse primers as follows: (5′-ACTCCTACGGGAGGCAGCAG-3′) and (5′-GGACTACHVGGGTWTCTAAT-3′). After recovery from agarose gel electrophoresis, three technical replicates of PCR products from each DNA sample were pooled, and the desired fragments underwent purification via the AxyPrep DNA Gel Extraction Kit (Axygen Biosciences, Axygen, USA). The purified fragments were quantified on a Quantus^™^ Fluorometer. Construction of sequencing libraries was performed using the NEXTFLEX^®^ Rapid DNA-Seq Kit. Thereafter, all libraries were accumulated in equimolar quantities prior to sequencing via the HiSeq 2500 system according to Illumina guidelines of Majorbio Co., Ltd. (Shanghai, China).

### ELISA

The serum concentrations of LPS, DAO, TNF-α, and IL-1β and the contents of PAP, ASMase, SMS, ATGL and HSL in colon tissue were quantified using ELISA kits. In short, following thaw at 4 °C, all serum and colon tissue samples were treated as directed by respective ELISA kits. Optical density was measured at 450 nm via an automatic microplate reader Mk3 (Thermo Fisher Scientific, USA).

### Analysis of short-chain fatty acids in serum and fecal samples

Sixty milligrams of each fecal sample collected in "Sample collection" section, and extracted with 300 μL of Milli-Q water under vortexing for 5 min, followed by a 10-min ultrasonic disruption in ice water. The extract was spun for 10 min at 14,000 rpm at 4 °C, and 200 μL supernatant was collected into a new Eppendorf tube for lipid extraction. Subsequently, the obtained supernatants were extracted with 50 μL of 50% H_2_SO_4_ and 200 μL of methyl-tert-butyl ether (MTBE) with 9.31 μg/mL of 2-methylvaleric acid as the endogenous standard. Following a 10-min vortex, the extract underwent a 10-min spinning at 14,000 rpm at 4 °C, and the resultant extract was placed in a clean glass vial for subsequent GC‒MS assessment.

Subsequently, 50 μL of individual serum samples were extracted with 50 μL 50% H_2_SO_4_ and 200 μL MTBE containing 0.31 μg/mL 2-methylvaleric acid as the internal standard. The extract underwent a 10-min vortex, then a 10-min centrifugation at 14,000 rpm at 4 °C. The resulting supernatant was placed in a clean glass vial for subsequent GC‒MS evaluation.

An Agilent 7890B gas chromatograph system attached to an Agilent 5975 mass spectrometer was used to perform GC‒MS analysis. The equipment contained a DB-WAX (30 m × 0.32 mm × 0.25 μm) capillary column. One millimeter of the lipid extract was inserted into split mode (1:1). Helium served as the carrier gas with 1.2 mL/min flow rate. The front inlet purge flow rate was 3 mL/min. The oven temperature was started at 95 °C for 1 min, increased to 180 °C at 10 °C /min, re-increased to 240 °C at 30 °C /min, and kept constant at this temperature for a 1-min period. The injection, transfer line, quad, and ion source temperatures were 250 °C, 250 °C, 150 °C and 230 °C, respectively. The electron ionization energy was 70 eV. Using the target ion, we determined the concentration of the analytes under the selected ion monitoring (SIM) mode and were confirmed by confirmative ions. Target ions (m/z) of valeric, butyric, propionic, acetic, 2-methylvaleric acid, and hexanoic acid were 45, 45, 60, 60, 74, and 60, respectively, following a solvent delay of 3 min.

### Targeted lipidomics analyses of serum by UPLC‒MS/MS

For targeted lipidomics analyses in serum samples, 10 μL serum was combined with 150 μL cold methanol which was, in turn, supplemented with standards as follows: PG (14:0/14:0) (100 ng/mL), PI (17:0/14:1) (100 ng/mL), FFA19:0 (100 ng/mL), FFA18:0-d3 (100 ng/mL), FFA16:0-d3 (200 ng/mL), TAG (15:0/15:0/15:0) (100 ng/mL), SM (d18:1/12:0) (15 ng/mL), Cer (d18:1/17:0) (20 ng/mL), PE (12:0/13:0) (400 ng/mL), LPC19:0 (15 ng/mL), and PC (19:0/19:0) (20 ng/mL). To extract lipids, the mixtures were vortexed for 30 s, mixed with 500 µL MTBE and then vortexed again for 20 min at room temperature (RT). Then, 125 µL Milli-Q water was introduced to samples, vortexed, then spun at 12,000 rpm at 4 °C for 10 min. Next, 300 µL supernatant underwent drying via a concentrator prior to resuspension in 100 µL of a water-containing solution: isopropanol: acetonitrile [5:30:65 (v/v/v)]. Samples were again agitated for 1 min, then spun as mentioned above. The resulting supernatants were evaluated using UPLC/MS–MS [17] (Supplementary Note 1.3).

### Spatial metabolomics analyses of liver and intestine tissues by DESI-MSI

Following cryosection preparation (15 μm) with Thermo Cryotome Fse (Thermo Ltd, USA), we mounted hepatic and intestinal tissue samples on glass microscope slides, prior to preservation at − 80 °C until further evaluation.

DESI-MSI was conducted via a Waters Xevo G2XS Q-TOF instrument (Waters, MA, USA) and a 2D DESI source (Prosolia, IN, USA). In this assay, 98% methanol with 0.1% formic acid and 98% methanol were set as spray solvents for the detection of liver and intestinal tissue cryosections in positive mode, respectively. In the negative mode, 98% methanol at 5 μL/min was set as a spray solvent for their detection. The DESI sprayer voltage was 4.5 kV, with nitrogen being delivered at 0.45 mPa from an external gas cylinder. Capillary voltages were adjusted to 4.5 kV (positive) and 4.0 kV (negative) modes. Pixel sizes (X and Y) were adjusted to 100 μm, providing a 100 μm spatial resolution. Mass spectra were acquired in positive and negative ion modes in the 50–1200 D range. High-Definition Imaging (v 1.4, Waters) and Masslynx (v 4.1, Waters) were used for data acquisition and mass spectrometry image processing. Images were initially normalized via total ion chromatography (TIC), after which each m/z images were displayed and overlaid. Then, we selected regions of interest (ROIs) for mass spectral extraction and additional analyses.

### Western blot

CT and ET of colon tissues were extracted as per directions from the protein isolation kit (GenePool/GPP1815), and protein quantification was done via the BCA Protein Assay Kit. Following denaturation, proteins were electrophoresed on SDS‒PAGE gels prior to transfer to PVDF membranes, with subsequent treatments with primary and secondary antibodies. Protein bands were visualized with a chemiluminescent reagent and quantification of target protein gray densities was completed with appropriate endogenous controls.

### Statistical analysis

Spearman’s rank correlation analyses employed the OmicStudio tools (https://www.omicstudio.cn). Partial least squares discrimination assessment (PLS-DA) via SIMCA-P (v16.0, Umetrics, Sweden) was performed to observe the clustering and trends of different treatment groups with the resulting mass data matrix of lipidomics analysis of endogenous metabolites. Permutation examinations were conducted (n = 200) to validate the PLS-DA model. Furthermore, an independent sample T test-based approach was employed for significantly differentially expressed metabolite identification among the four groups. The distinct abundance of the resulting metabolites of interest was confirmed between groups using SPSS (v26.0) (IBM, Chicago, USA). All data are presented as mean ± standard deviation (SD). The Wilcoxon test was performed to compare microflora abundance in different treatment groups on the www.majorbio.com platform using 16S rDNA sequencing data. *p* ≤ 0.05 by FDR adjustment was set as the significance cut-off.

## Results

### Chemical basis and the active serum prototype components of SHTB

The respective base peak intensity (BPI) chromatograms for SHTB acquired in ESI^+^ and ESI^−^ modes are shown in Fig. 1A, B. Compound identification was completed via comparison of high-resolution mass spectra and MS/MS spectra to standard compounds and published information. Consequently, 210 compounds were discovered, including anthraquinones, anthrones, stilbene glycosides, amino acids, terpenoids, flavonoids, and coumarins, among others (Table S4).

Based on screening results and frequently reported bioactivities, anthraquinones and naphthopyranone were selected for further quantification analysis with method validation (Supplementary Note 1.2). In total, 21 main ingredients were quantified (Table 1). The average contents of different ingredients in SHTB ranged from 0.48 mg/kg (chrysophanol) to 531.31 mg/kg (emodin-8-O-β-D-glucopyranoside). Each ingredient had the same order of magnitude of amount in different batches of SHTB.

**Table 1:** The contents of 21 main ingredients in different batches of SHTB (mean ± SD, n = 6)

Analyte	Content (mg/kg)						
	26200212	26210152	26200133	26210123	26200052	26210133	(Mean ± SD)
Cassiaside B2	61.50	63.36	47.14	50.54	58.48	62.50	57.25 ± 6.80
Chrysophanol 1-O-β-triglucoside	195.03	196.49	172.21	170.61	159.17	188.98	180.41 ± 15.24
Cassiaside C	399.12	405.18	362.23	341.04	335.20	372.09	369.14 ± 28.97
Chrysophanol-1-O-β-gentiobioside	75.03	70.93	61.79	60.99	59.42	68.01	66.03 ± 6.26
Cassiaside B	39.74	40.02	35.13	33.51	32.70	36.53	36.27 ± 3.09
Aurantio-obtusin beta-D-glucoside	182.30	176.49	158.41	155.63	118.59	166.53	159.66 ± 22.57
Physcion 8-O-β-glucopyranoside	134.11	136.27	117.90	117.73	113.84	120.27	123.35 ± 9.43
Emodin-8-O-β-D-glucopyranoside	568.95	565.14	516.47	504.18	498.73	534.38	531.31 ± 30.29
Aloe-emodin-glucoside	2.62	2.79	2.57	2.30	2.34	2.53	2.52 ± 0.18
Cassiaside	107.23	98.65	93.23	96.15	95.76	94.15	97.53 ± 5.11
Chrysophanol 8-O-glucoside	1.01	1.38	1.27	1.09	0.54	0.67	0.99 ± 0.33
Chrysoobtusin	54.25	54.80	51.39	50.59	48.86	52.71	52.10 ± 2.26
Obtusin	38.33	38.90	35.62	34.14	32.83	37.43	36.21 ± 2.42
Aurantio-obtusin	159.5	163.39	142.67	141.15	137.83	155.79	150.06 ± 10.80
2-hydroxyl emodin-1-methyl ether	13.52	13.42	12.43	11.97	12.14	12.73	12.70 ± 0.65
Physcion	74.27	77.37	69.97	65.78	64.24	68.29	69.99 ± 5.03
Rhein	8.37	8.58	7.66	7.61	7.77	7.71	7.95 ± 0.42
Obtusifolin	85.46	86.53	78.63	75.98	76.53	80.83	80.66 ± 4.49
Emodin	267.99	270.01	251.69	245.60	248.82	259.43	257.26 ± 10.2
Aloe-emodin	556.09	552.78	519.18	489.28	509.04	519.42	524.30 ± 25.81
Chrysophanol	0.47	0.50	0.42	0.51	0.34	0.63	0.48 ± 0.10

To conduct an initial material-based exploration focused on the mechanisms through which SHTB can improve the symptoms of STC, the active serum components of SHTB were analyzed by collecting serum samples from rats that had been treated with this TCM preparation. This approach facilitated the identification of 21 serum prototype components of SHTB (Table S5).

### SHTB relieves diphenoxylate-induced STC

Diphenoxylate-treated rats were used as study models to assess the therapeutic effects of SHTB. The duration between a charcoal meal and the first black stool (intestinal transit time) as well as fecal water content were utilized for evaluation of STC regulation in rats. Diphenoxylate-treated rats developed constipation, evidenced by the prolonged transit duration and reduced water amount in feces at 5 h and 12 h (Fig. 2B), compared to the saline-administered controls. SHTB administration strongly relieved diphenoxylate-triggered constipation, and SHTB could promote gastrointestinal peristalsis and defecation, as evidenced by reduced transit duration and enhanced water amount in fecal samples.

### SHTB treatment augments intestinal barrier function and alleviates systemic inflammation in a rat model of STC

Previous studies suggest that LPS can open the intestinal barrier, causing increased intestinal permeability and promoting gut inflammation when the gut is damaged at low sIgA levels [18]. The increase in serum DAO suggests intestinal barrier disruption [19, 20]. DAO and LPS levels in serum can act as an important index to evaluate intestinal barrier activity and as a predictor of the occurrence and development of inflammation. Additionally, the intestinal inflammation induced by TNF-α receptor signaling in intestinal epithelial cells promotes intestinal barrier disruption [21]. Herein, we demonstrated increased circulating concentrations of LPS and DAO in the model group (Fig. 2C). SHTB restored circulating contents of LPS and DAO (*p* < 0.05, Fig. 2C). In addition, SHTB intake reduced the circulating concentrations of two inflammatory cytokines (TNF-α and IL-1β) relative to model rats (*p* < 0.05, Fig. 2C). Thus, these results indicate that SHTB administration could block serum levels of inflammatory cytokines, improve intestinal barrier damage and increase intestinal motility. Correlation coefficients between active serum prototype components of SHTB and fecal water contents at 5 h and 12 h, transit duration of the first black stool, LPS, DAO, and inflammatory factors (TNF-α and IL-1β) were determined to determine the chemical constituents of SHTB responsible for its therapeutic effects in attenuation of diphenoxylate-induced STC. Significant correlations are shown in Fig. 2D. Fecal water contents at 5 h and 12 h positively correlated with active serum prototype components, including ginsenoside Rb1, aloins and obtusin; LPS, TNF-α and IL-1β were negatively correlated with chrysophanol, rhein and ginsenoside Rb2/Rb3, respectively.

### SHTB remodeled the intestinal bacteria of STC rat models

**Fig. 3:**
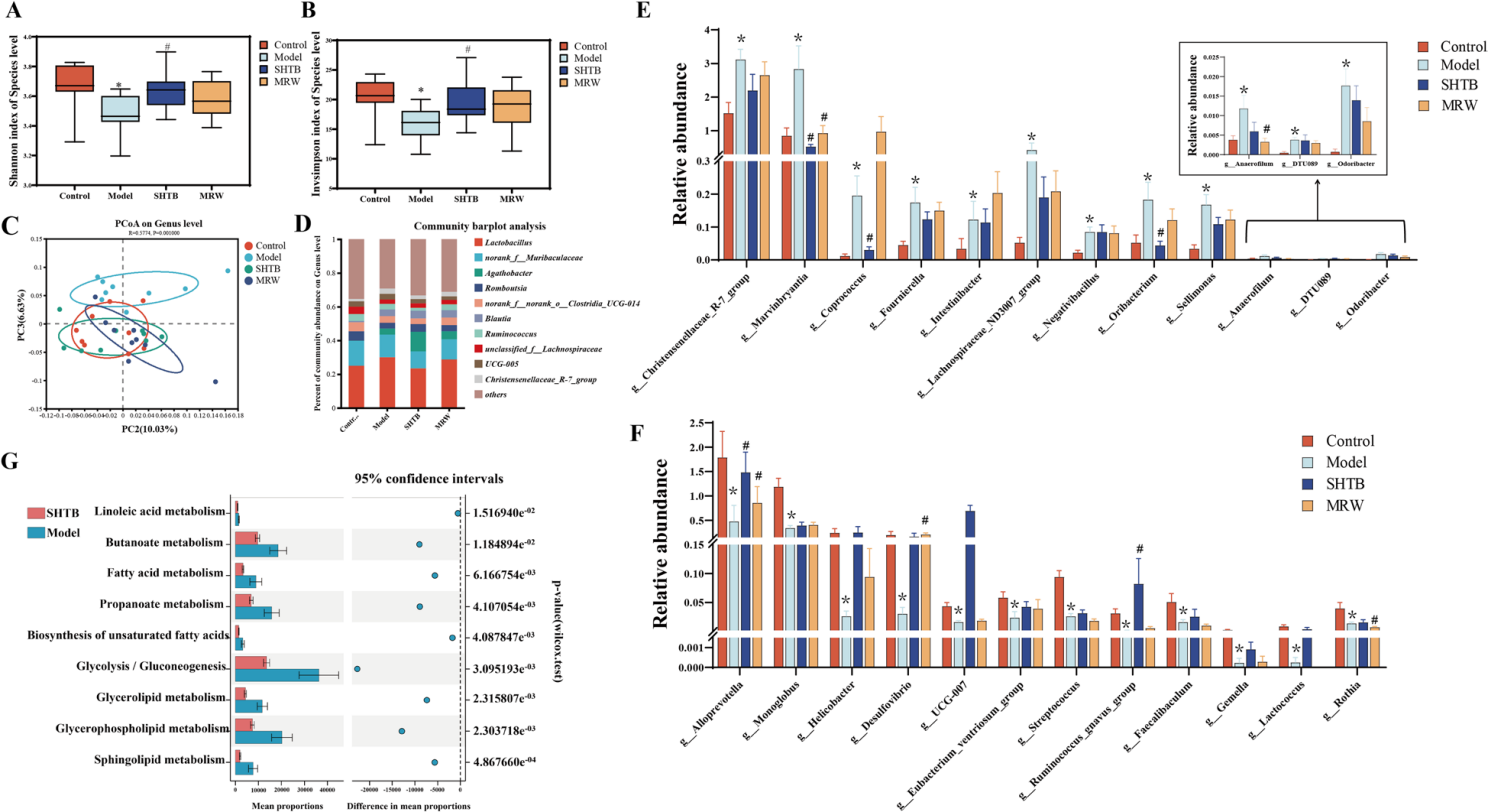
The shift of gut microbiota in different groups according to the 16S rDNA data. (A-B) The estimate of **A** Shannon and **B** invsimpson index analysis in different groups at the species level. **C** Principal coordinate analysis (PCoA) of the microbiota based on the unweighted_unifrac distance metrics for different groups. **D** The relative gene-level abundance of different members of the intestinal microbiota in these four groups. **E**, **F** The increased relative abundance of microbial species **E** and the decreased relative abundance of bacterial species **F** at the genus level. **G** Functional prediction of gut microbiota between the model and SHTB groups. Data in **A** and **B** were analyzed with Wilcoxon rank-sum tests and are presented as medians with interquartile ranges, minimums, and maximums. Data in **E** and **F** were analyzed with Wilcoxon rank-sum tests and are presented as the mean ± SD. n = 9 per group. Control is the control group; the model is the constipation model group; SHTB is the treatment group with the SHTB group; MRW is the treatment group with the MRW group. * represents the difference between the control and model groups (*p < 0.05; **p < 0.01); # represents the difference between the model and SHTB groups (#*p* < 0.05)

The gut microbiota mediates gastrointestinal homeostasis and activities. To investigate the SHTB influence on gut microbiota composition, 16S rDNA sequencing was performed using the Illumina HiSeq platform. As shown in Figs. 3A and B, species diversity was drastically lower in model versus control and SHTB rats, as revealed by the Shannon and InvSimpson indices. The unweighted UniFrac distance matrix-based principal coordinates analysis (PCoA) identified marked variations at the genus level in microbial composition. The model rats was split from controls, suggesting a significant change in gut microbiota composition in model rats (Fig. 3C). The SHTB and MRW groups were in close proximity to control rats, indicating that SHTB administration can potentially restore gut microbiota composition (Fig. 3C). Thereafter, we examined the gut microbiota composition at the genus taxonomic level. Figure 3D presents genus-based alterations in microbiota composition. At the genus level, the higher community population of the sample was *Lactobacillus, norank_f_Muribaculaceae, Agathobactor, and Romboutsia* (Fig. 3D). The Wilcoxon examination compared microflora relative abundance between various treatments. Unlike controls, the relative abundances of *Christensenellaceae_R-7_group, Marvinbryantia, Coprococcus, Fournierella, Intestinibacter, Lachnospiraceae_ND3007_group, Negativibacillus, Oribacterium, Sellimonas, Anaerofilum, DTU089,* and *Odoribacter* were markedly increased in the model group (Fig. 3E), whereas the relative abundances of *Alloprevotella, Desulfovibrio, Eubacteriumventriosumgroup, Faecalibaculum, Gemella, Helicobacter, Lactococcus, Monoglobus, Rothia, Ruminococcusgnavus group, Streptococcus,* and *UCG-007* were drastically reduced in model rats (Fig. 3F). Following SHTB administration, all these bacteria were improved; among them, SHTB intake significantly regressed *Alloprevotella, Coprococcus, Marvinbryantia, Oribacterium,* and *Ruminococcus gnavus* to control group levels (*p* < 0.05). These findings revealed that SHTB administration can restore the intestinal bacterial composition and structure at the genus taxonomic level.

In addition, the gut microbiota possesses multiple metabolic activities. A detailed understanding of the intestinal microbial activity will enhance our knowledge of the relevant signaling networks behind SHTB action on STC. In this report, metabolic gut microbial actions were estimated via 16S rDNA sequencing data-based PICRUSt2. In all, nine metabolic networks were strongly influenced among the control, model, and SHTB groups, including linoleic acid, butanoate, fatty acid, propanoate, biosynthesis of unsaturated fatty acid, glycolysis/gluconeogenesis, glycerolipid, glycerophospholipid, and sphingolipid metabolisms. Unlike the model rats, the relative abundance of a total of nine metabolic pathways decreased in the control and SHTB rats (*p* < 0.05, Fig. 3G). These findings show that SHTB administration can potentially modulate intestine microbial activity in constipated rats.

### SHTB controls SCFAs formation and metabolism

**Fig. 4:**
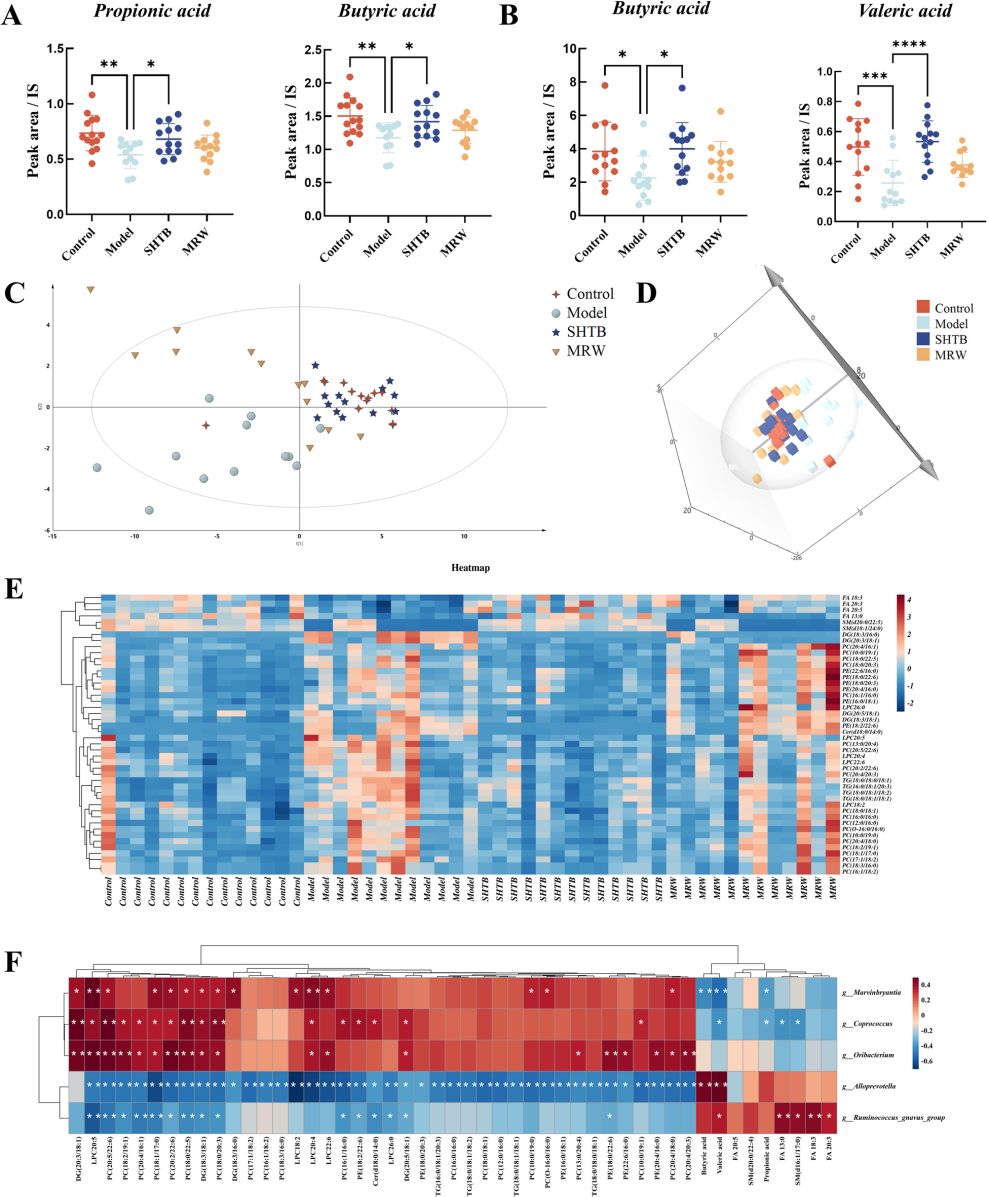
Effect of SHTB on endogenous metabolites in the feces and serum of rats. **A** The relative concentrations of butyric acid and valeric acid in fecal samples **B** The relative concentrations of propionic acid and butyric acid in the serum samples. **C** 2D and **D** 3D score plots of the PLS-DA model using the lipidomics data from individual serum samples. **E** Heatmap of potential biomarkers of SHTB treatment based on targeted fatty acid and lipid analysis. **F** Heatmap of correlation analysis between differential genus microbiota and differential lipid metabolites. Data are means ± SDs and were compared using Student’s t tests. n = 8–14 per group. *p < 0.05; **p < 0.01; ***p < 0.001; ****p < 0.0001

SCFAs, a major group of fermentation metabolite dietary fibers that are produced by anaerobic intestinal microbiota, beneficially affect mammalian energy metabolism [22, 23]. SCFAs are strongly associated with intestinal peristalsis and inflammation. The SCFAs in fecal and serum samples were evaluated to check their levels in the gastrointestinal tract and systemic circulation, respectively. Five SCFAs (i.e., valeric, butyric, propionic, acetic, and hexanoic acids) were detected in both fecal and serum samples of rats from all four groups. The relative amounts of SCFAs in fecal samples were in the order butyric acid > acetic acid > valeric acid > propionic acid > hexanoic acid. The SCFA with the highest relative abundance in serum samples was acetic acid, followed by butyric, propionic, hexanoic, and valeric acids. The diphenoxylate-induced model group exhibited significantly lower contents of butyric, valeric, and propionic acids than controls (Fig. 4A, B). However, SHTB-treated rats exhibited significantly elevated levels of butyric, valeric, and propionic acids (*p* < 0.05 by FDR adjusted) relative to the model group. Differences in the SCFA contents between control and SHTB rats were insignificant. These results indicate that SHTB corrected the secretion of particular SCFAs in rats with diphenoxylate-induced STC.

### SHTB improved lipid metabolism in STC rat models

The gut microbiota potentially influences host health by modulating host metabolism. Herein, nine metabolic networks were strongly impacted among the various rat groups based on PICRUSt2 analysis and primarily involved fatty acids as well as lipid metabolism. Therefore, lipidomics was used to investigate alterations in circulating metabolome, which elucidated potential signaling mechanisms behind the SHTB-mediated regulation of gut microbiota and constipation. Serum lipidomics was performed to screen for lipids using UPLC‒MS/MS. The collected mass data were subjected to PLS-DA to explore the serum lipid metabolic profiles of the four rat groups and identify novel biomarkers. In Figs. 4C and D, the control and SHTB groups were entirely separated from the model group, indicating that the lipid metabolic states of STC rats differed from the control and SHTB rats. Moreover, unlike the model rats, metabolites from the SHTB rats were distributed closer to the control rats, demonstrating that lipid metabolic states of STC rats were beneficially regulated by SHTB. Using permutation analysis, we next verified the stability and reproducibility of the PLS-DA model (n = 200) (Figure S1).

Through statistical analysis, SHTB corrected forty-six dysregulated endogenous lipids in constipation (*p* < 0.05 by FDR adjusted) (Fig. 4E). Notably, administration of SHTB increased the levels of long-chain fatty acids (FA 13:0, FA 18:3, FA 20:3, and FA 20:5) and sphingomyelins (SM(d20:0/22:4) and SM(d16:1/17:0)) and reduced 5 lysophosphatidylcholines, 20 phosphatidylcholines, 1 ceramide, 6 phosphatidylethanolamines, 4 triglycerides, and 4 diglycerides relative to model rats (Fig. 4E). Next, we conducted correlation analysis to screen for therapeutic biomarkers related to SHTB pharmacodynamic indicators and inflammatory factors. As shown in Fig. 4F, the correlation analysis between key discriminative microbiota and improved endogenous lipid biomarkers by SHTB showed that *Alloprevotella* and *Ruminococcus_gnavus* were negatively correlated with most lipid differentially abundant metabolites, *Alloprevotella* was positively correlated with SCFAs (butyric and valeric acids), and *Ruminococcus_gnavus* was significantly and positively correlated with long-chain fatty acids (FA 13:0, FA 18:3 and FA 20:3) and valeric acid. *Coprococcus*, *Marvinbryantia* and *Oribacterium* were significantly correlated with most lipid differentially abundant metabolites, including LPCs, PCs, ceramide, etc. *Marvinbryantia* was inversely linked to SCFAs (propionic, butyric, and valeric acids), and *Coprococcus* was significantly and inversely correlated with propionic acid, valeric acid, FA 13:0, and SM (16:1/17:0).

### SHTB regulated metabolic disorders in the liver and intestines of STC rat models

**Fig. 5:**
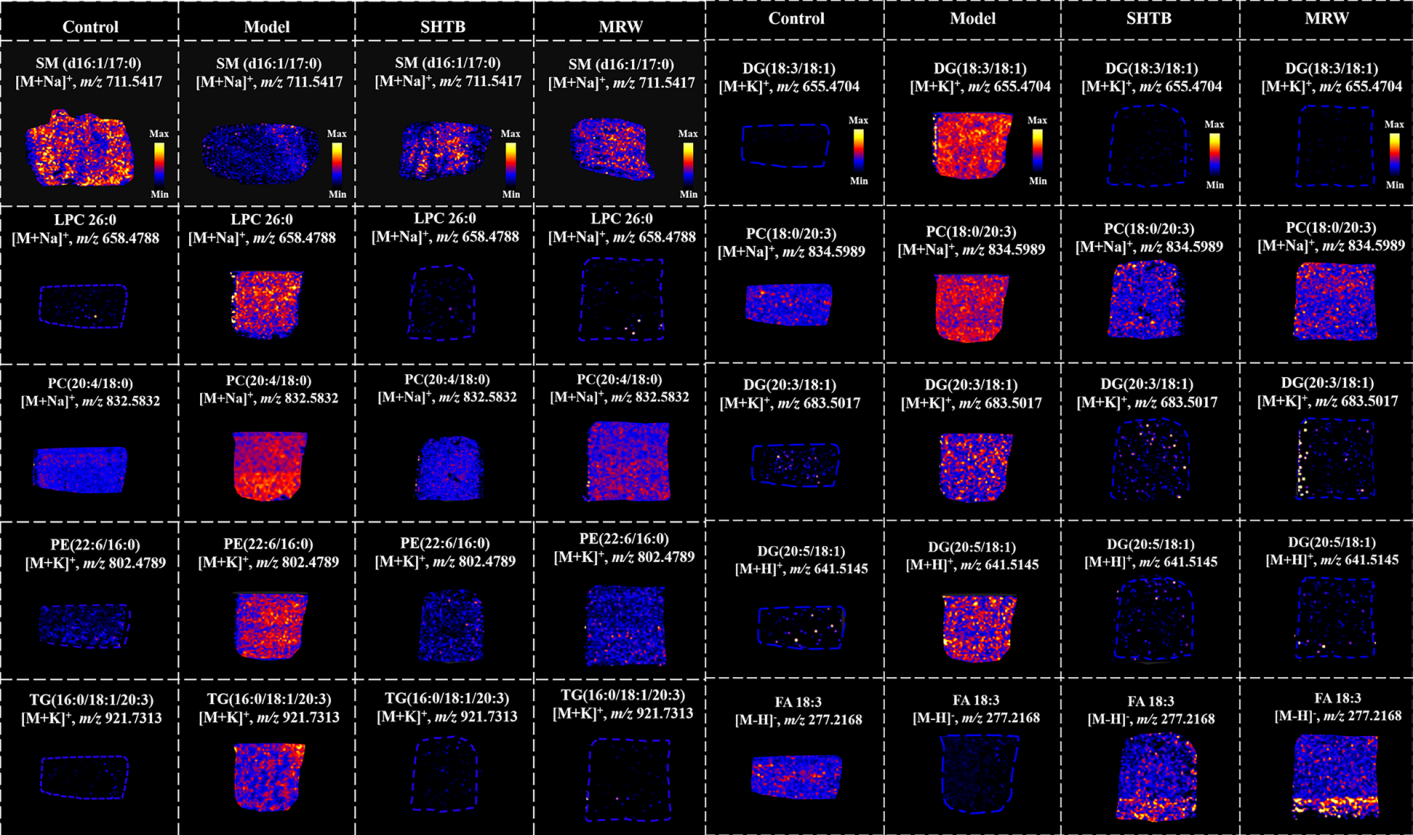
Differentially abundant metabolites from liver and intestine tissue samples by spatial metabonomics. SM(d16:1/17:0) from intestinal tissue imaging results; other results are derived from liver tissue imaging data

Three ROI for individual liver and intestinal section were chosen, and raw ion intensity information were retrieved for t test analyses. The DESI results showed that the levels of some lipids in the liver were consistent with those in serum from the four groups. The levels of LPC 26:0, PC (20:4/16:1), PC (13:0/20:4), PE (22:6/16:0), TG (16:0/18:1/20:3), DG (18:3/18:1), DG (18:3/16:0), and DG (20:5/18:1) were upregulated in STC model rats and markedly downregulated following SHTB treatment. FA 18:3 exhibited the opposite trend; they were reduced in the STC model rats and increased in the SHTB rats, in tandem with findings from serum samples (Fig. 5). In intestinal tissues, SM (d16:1/17:0) was significantly elevated after SHTB administration relative to the model rats, and this trend corroborated with the serum sample results (Fig. 5).

### Analysis of key enzymes for regulating differentially expressed metabolites

**Fig. 6:**
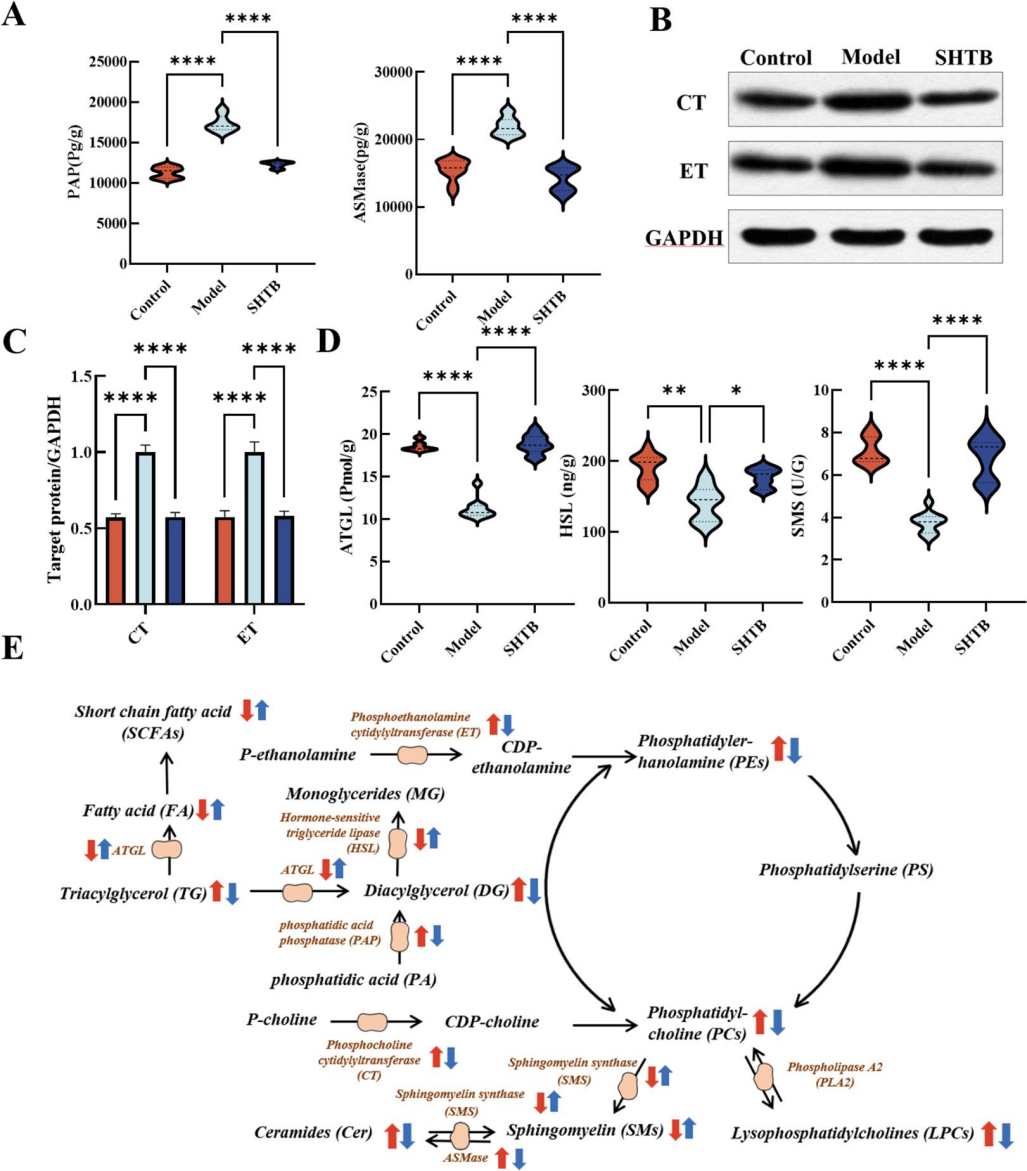
**A** The levels of PAP and ASMase in colon tissue were detected by ELISA kits. **B**, **C** The levels of CT and ET in colon tissue were detected by western blot (n = 3). **D** The levels of HSL, SMS and ATGL in colon tissue were detected by ELISA kits. **D** Potential STC and SHTB-related biomarker networks. Arrows (“↑↓”) indicate metabolites that were significantly up- and downregulated in the model (red) relative to controls. Blue shows the metabolites that were significantly altered in the SHTB group relative to the model group. *p < 0.05; **p < 0.01; ****p < 0.0001

According to the lipidomics results, we focused on some key enzymes in the lipid metabolism pathway, including ET, CT, PAP, ASMase, SMS, ATGL and HSL. We measured the protein expression of colon ET, CT, PAP, ASMase, SMS, ATGL and HSL by molecular biological analysis. Our analysis revealed that the colon ET, CT, PAP and ASMase contents of STC rats were significantly increased, and SMS, ATGL and HSL were significantly decreased. SHTB abrogated the accumulation of colon ET, CT, PAP and ASMase of STC rats (Fig. 6A–C) and significantly increased colon SMS, ATGL and HSL expression of STC rats (Fig. 6D).

## Discussion

In China, SHTB is an herbal formula used to treat STC, yet its chemical constituents and potential mechanisms of action have not been fully studied, particularly from the perspectives of lipidomics and intestinal flora. This study established a diphenoxylate-induced STC rat model and selected MRW as a positive control due to its regulatory impact on intestinal microflora structure and improving the metabolism of SCFAs [24]. SHTB attenuated intestinal barrier permeability and the inflammatory response by promoting the production of SCFAs, inhibiting lipid accumulation, regulating relevant enzyme expressions in lipid metabolism, and restoring intestinal flora homeostasis, hence alleviating STC.

This work conducted a systematic and comprehensive identification of chemical components using UPLC-Q-TOF–MS technology. Consequently, 210 compounds were identified, including anthraquinones, anthrones, stilbene glycoside, ginsenoside, amino acids, terpenoids, flavonoids, and coumarins. The levels of 21 main anthraquinone and naphthopyranone active ingredients in SHTB ranged between 0.48 and 531.31 mg/kg, providing novel insights for the quality control of SHTB. Additionally, we also detected blood-entering components of SHTB. Although the chemical composition of TCM is complex and varied, its clinical efficacy is largely dependent on the levels of effective components [25]. The major constituents of *Polygonum multiflorum*, *Aloe vera,* and *Semen cassiae* in SHTB include anthraquinones and naphthopyranone with purgative effects [26–30], most of which are glucosides. Free anthraquinones include chrysophanol, aloe emodin, emodin, rhein, etc. [29–31]. Emodin, aloe-emodin, and chrysophanol have antiviral, anti-inflammatory, antibacterial, and neuroprotective activities and thus are possible therapeutic options for prophylaxis and constipation management [32–34]. In addition, rhein can increase the contractile activities of the cecum and increase the speed of contraction of the circular muscle of the colon, helping transport intestinal contents [35]. Rhein can enhance motor activity and colonic electromyography in constipated mice and reduce AQP3 expression in the colonic mucosa, thereby efficaciously alleviating constipation symptomology [36]. Most anthraquinone compounds enter the large intestines to stimulate the nerve plexus in the submucosa and muscular layer and promote colonic peristalsis [37]. Therefore, it is important to quantify the effective components of Chinese medicines [38]. These data indicate that SHTB administration exerts beneficial effects due to the combined effect of multiple components.

Increasingly, studies have shown that animals and patients with constipation have severe enteric dysbacteriosis, whereas the reconstruction of the intestinal microbial community effectively relieves constipation symptoms [8, 39]. Herein, we employed 16S rDNA gene sequencing to examine the species diversity (alpha and beta diversity) and differential genera of intestinal microflora in STC rat models treated with SHTB. Alpha diversity analysis revealed that the intestinal species diversity of STC rat models was markedly lower relative to the control group. Beta diversity analysis also revealed that constipation affects intestinal species diversity, consistent with other findings [40]. In addition, the signaling networks of SHTB in STC therapy may involve regulating the relative abundance of *Alloprevotella*, *Coprococcus*, *Marvinbryantia*, *Oribacterium,* and *Ruminococcus gnavus*. An increase in the abundance of *Alloprevotella* promotes excretion in constipated mice [41], potentially by increasing the abundance of SCFAs and promoting TPH1 as well as 5-HT synthesis [42]. *Coprococcus* correlates with isovalerate and butyrate production in human intestines, and its abundance tends to be increased in constipated patients [43]. In addition, *Ruminococcus gnavus* was one of the first stomach bacteria to be discovered with beneficial effects, including stabilizing the intestinal barrier, reducing kidney stones, and improving energy circulation [44, 45]. Furthermore, *Marvinbryantia* belongs to *Firmicutes* [46]. A prior investigation found that the relative population of *Firmicutes* were strongly upregulated in the constipation group based on phylum analysis [47]. *Marvinbryantia* was also positively related to TNF-α and negatively correlated with intestinal barrier-tight junction proteins in high-fat diet-fed rabbits [48]. The *Oribacterium* population is positively related to the colonic barrier permeability, which accelerates colon inflammation and is a potentially pathogenic bacterium [49]. We demonstrated that SHTB can be used to treat constipation by increasing the abundance of *Ruminococcus gnavus* and *Alloprevotella* while reducing *Coprococcus*, *Marvinbryantia* and *Oribacterium*. Furthermore, functional prediction was assessed using 16S rDNA sequencing. The results revealed that metabolic networks, such as, linoleic acid metabolism, butanoate metabolism, fatty acid metabolism, propanoate metabolism, biosynthesis of unsaturated fatty acids, glycolysis/gluconeogenesis, glycerolipid metabolism, glycerophospholipid metabolism, and sphingolipid metabolism, were significantly influenced by SHTB intervention. This suggests that imbalances in gut microbiota closely correlate with constipation. Based on the above, we speculate that the SHTB capsule can regulate STC-induced disorders of the gut microbiota, which may be linked to the inflammatory response, intestinal barrier integrity and lipid metabolism.

To evaluate these conjectures, we assessed the expression of gut barrier injury-related indicators and cytokines in these four groups. The results revealed that SHTB could reduce the LPS, DAO, and inflammatory factor levels in serum. These therapeutic effects were significantly correlated with ferulic acid, aloesin, formononetin, chrysophanol, and daidzein (Fig. 2D). Previous reports indicate that DAO is a prolific cytoplasmic enzyme found within villous cells in the upper layer of the intestinal mucosa and a marker for assessing gut barrier function. When the gut barrier is impaired, DAO enters the blood, and Ich increases serum DAO [21]. Therefore, changes in serum DAO content are closely related to gastrointestinal mucosal barrier functional impairment [50]. In addition, impaired intestinal barrier permeability could promote LPS leakage into the circulatory system, causing inflammatory responses and evoking gastrointestinal motility disturbances as well as oxidative stress [51, 52]. Furthermore, the bacterial endotoxin LPS can regulate gastrointestinal motility by inhibiting sphincter function or increasing intestinal transit time. A recent study found a positive correlation between serum endotoxin activity and constipation in chronic hemodialysis patients [53]. Additionally, intrusion of inflammatory cells into the digestive tract is a critical histological alteration that modulates gastrointestinal motility disorders [54]. Emerging evidences revealed that constipated rats display a remarkable level of colonic inflammatory response, and anti-inflammatory aid in relieving constipation among these rats [55]. Consistently, the STC model rats in this study had significantly increased serum TNF-α and IL-1β levels, while treatment with SHTB significantly suppressed the production of these proinflammatory cytokines, which mediate constipation progression. These data suggest that SHTB reduced the translocation of LPS and inflammatory factors into the systemic circulation by restoring the intestinal barrier integrity, hence improving intestinal flora disturbance and constipation.

According to the results of gut microbiota analysis and functional prediction, metabolomics was performed in serum and feces, and MSI was used in liver and intestinal tissues to screen for lipids related to STC and SHTB treatment mechanisms. Our research results revealed that, unlike the model group, the propionic, butyric, and valeric acid levels were upregulated in the SHTB-treated rats (*p* < 0.05, Fig. 4A, B). In total, 46 different metabolites, including LPCs, PCs, SMs, ceramides, PEs, TGs, DGs, and fatty acids (FA 13:0, FA 18:3, FA 20:3, and FA 20:5), displayed a significant regression trend in SHTB-treated STC rats. Functionally, SCFAs can influence intestinal mucosa integrity, regulate glucose metabolism, lipid metabolism, the immune system, and inflammatory reactions, induce the differentiation of colon regulatory cells (Tregs), and improve intestinal barrier functions [56]. At the same time, our correlation analysis confirmed that among the bacteria with remarkable differences between the sham and model rats, *Alloprevotella* had positive associations with valeric acid. Additionally, SCFAs had negative associations with *Coprococcus* and *Marvinbryantia* (*p* < 0.05, Fig. 4F). However, both *Coprococcus* and *Marvinbryantia* are important producers of SCFAs; we hypothesized that SCFAs decreased in model rats, resulting in a compensatory increase in *Coprococcus*. *Marvinbryantia* caused a negative correlation between *Coprococcus*, *Marvinbryantia,* and SCFAs.

Moreover, constipation is associated with dyslipidemia; lipid accumulation may result in impaired integrity of the intestinal barrier, organelle dysfunction, cell injury, and chronic inflammation in vivo [57, 58]. In our study, 5 LPCs, 20 PCs, 6 PEs, 4 DGs and 4 TGs significantly increased in the STC model rats and strongly decreased following SHTB treatment (Fig. 4E). In addition to disturbance of LPCs, PCs, TGs and DGs in the model group, normal metabolism of Cers, SMs and fatty acids (FA) was also disrupted. All nucleated mammalian cells generate PC via the CDP-choline Kennedy pathway [59, 60]. Choline kinase phosphorylates choline to form phosphocholine, which is then transformed by CTP-phosphocholine cytidylyltransferase (CT) to CDP-choline. Then, CDP-choline: 1,2-diacylglycerol cholinephosphotransferase accelerates PC production from CDP-choline [61]. PC was hyperactivated in STC model rats and may regulate the expression of CT, thereby affecting lipid metabolism and oxidation. Correspondingly, PE was produced by the CDP-ethanolamine pathway. Phosphoethanoamine (P-ethanolamine) to synthesize CDP-ethanolamine with help from the cytoplasmic enzyme CTP: phosphoethanolamine cytidylyltransferase (ET). CDP-ethanolamine:1,2-diacylglycerol ethanolaminephos photransferase transforms CDP-ethanolamine and diacylglycerol to PE (Fig. 6E). Herein, we evaluated CT and ET to examine the modulatory network of STC and SHTB action on STC. We demonstrated that STC modeling produced a remarkable upregulation of colon CT and ET, whereas SHTB treatment downregulated colon CT and ET in STC rats. Based on these findings, CT and ET are the key targets of SHTB in regulating the synthesis of PC and PE to relieve constipation. In addition, phospholipase A2 (PLA2) accelerates LPC production from PC [62] (Fig. 6E). As a functional lipid, LPCs participate in the regulation of cell proliferation and invasion [63] and can promote inflammation [64] as well as increase vascular endothelial permeability [65]. In addition, PC and ceramides generate SMs under catalysis by SM synthase (SMS). SMs are essential lipids of intestinal epithelial cells that maintain intestinal barrier integrity, regulate nutrient uptake, and modulate intestinal mucosa differentiation and regeneration [66]. Furthermore, ceramides is also formed via SM and glycosphingolipids hydrolysis by acid sphingomyelinase (ASMase) within the Golgi (Fig. 6E) [67]. Ceramides are key molecules of sphingolipid metabolism that can influence insulin resistance, oxidative stress, inflammation, apopto sis, and other important life processes [68, 69]. Furthermore, ceramides in intestinal epithelial cells can increase the activities of NF-kB by suppressing IkB-a and IkB-b protein levels [70, 71]. Overexpressed proinflammatory molecules also destroy connexins such as Claudin-1, which is essential for maintaining the structural integrity of intestines [72]. During STC, TNF-α, IL-1, and other inflammatory properties can activate sphingomyelinase (SMase) to increase ceramide levels [73]. Therefore, constipation is usually accompanied by cIlon inflammation and intestinal damage [74, 75]. Our study showed that the changes in SMS and ASMase in STC rats involved in SM and ceramide metabolic pathways were largely reversed by SHTB treatment (Fig. 6A and D).

**Fig. 7:**
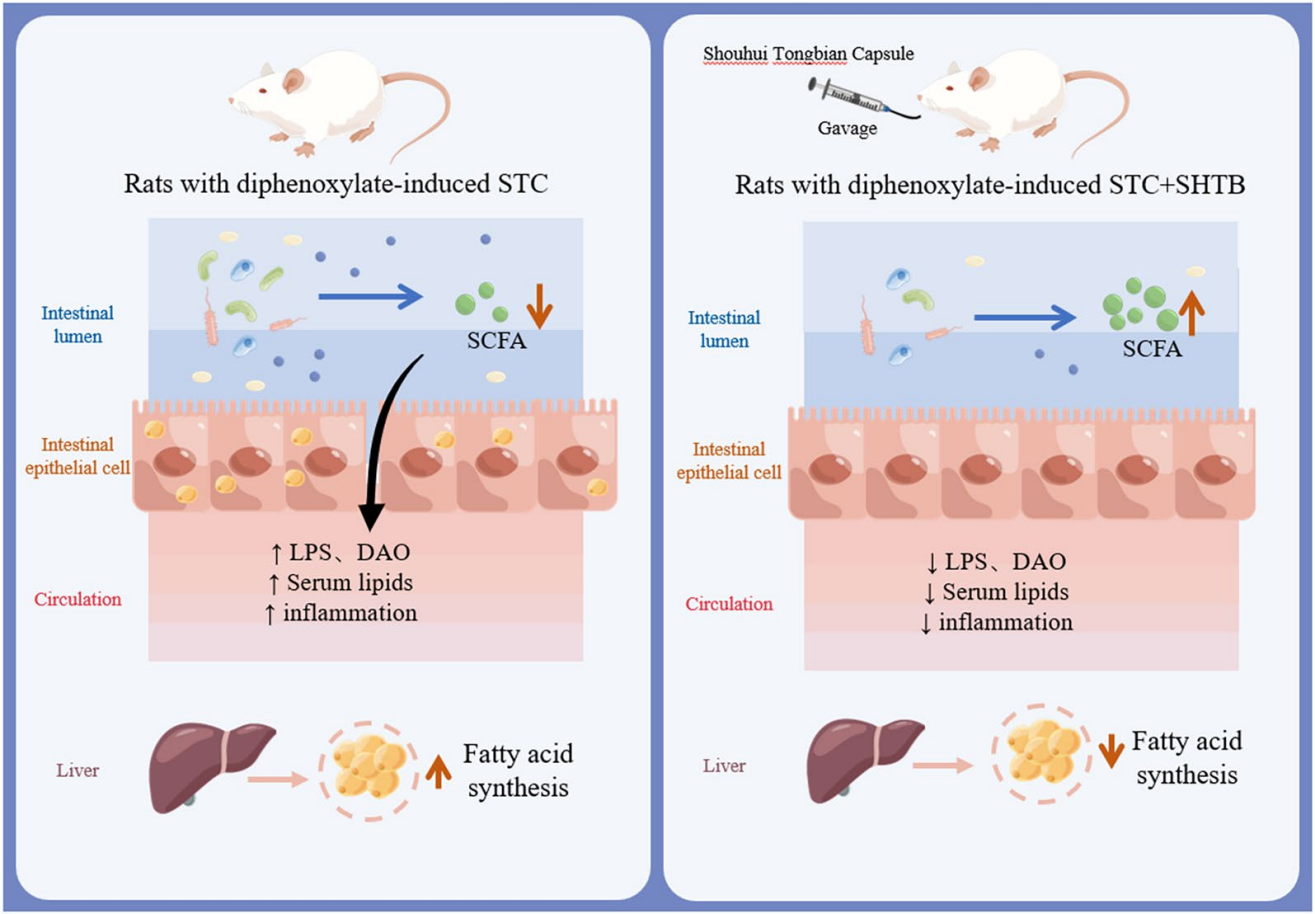
Schematic diagram of the potential mechanism by which SHTB alleviates STC in rats

Fatty acid mobilization from triglyceride stores within adipose tissue necessitates lipolytic enzymes. ATGL and HSL hydrolyze triglycerides within the mammalian adipose tissue [76]. A highly cited publication from 1964 reported that HSL had a higher level of diglyceride hydrolase, but not triglyceride hydrolase activity [77]. Emerging evidences confirm that ATGL is critical for the beginning stage of lipolysis among human adipocytes. Moreover, HSL is rate-limiting for diglyceride catabolism. ATGL catalyzes the hydrolysis of TG to DG and FA, and then HSL degrades DG to monoglycerides (MG) [76, 78, 79]. Moreover, phosphohydrolase (PAP) could convert phosphatidic acid (PA) into DG (Fig. 6E) [80]. It is the key enzyme regulating the content of DG in organisms. Aberrant lipid metabolism is a major factor in metabolic disease etiology. Augmented intestinal fat absorption contributes to inflammatory diseases namely inflammatory bowel disease (i.e., ulcerative colitis and constipation) [81, 82]. SHTB capsules can reverse the high expression of ATGL, HSL and PAP in STC rats, inhibit the synthesis of DG and promote the decomposition of TG and DG to relieve colonic lipid accumulation in STC rats and improve constipation. FA in patients with gastrointestinal diseases of constipation and diarrhea is significantly decreased [83], and intestinal FA deficiency is a major characteristic of constipation [84], which is consistent with our findings. In summary, the metabolic profiles of the liver and intestines are significantly altered by constipation. Changes in liver and intestinal metabolites in STC rat models after SHTB administration were primarily associated with key enzymes of fat mobilization and metabolism, such as CT, ET, PAP, ASMase, SMS, ATGL and HSL. Moreover, these metabolites exhibited comparable expression trends in both tissue and serum samples. Therefore, they are potential biomarkers and treatment targets for constipation. Figure 6E shows the potential STC and SHTB-related biomarker networks. Herein, SHTB consumption attenuated intestinal barrier permeability and the inflammatory response by promoting the production of SCFAs, suppressing lipid accumulation, and restoring intestinal flora homeostasis, hence alleviating STC (Fig. 7).

## Conclusions

In conclusion, our study identified 210 compounds and quantified 21 major active ingredients from SHTB to establish the chemical basis of SHTB. After qualitative analysis of SHTB blood components, we examined the signaling pathways associated with the SHTB-mediated regulation of constipation with particular focus on gut microbiome and metabolome. SHTB reduced the circulating LPS, DAO, TNF-α, and IL-1β contents, thus inhibiting the inflammatory response and restoring the intestinal barrier integrity. In addition, SHTB regulated the relative abundances of *Alloprevotella, Coprococcus, Marvinbryantia, Oribacterium,* and *Ruminococcus gnavus* to reshape the structure and function of the intestinal flora. Functional prediction revealed that lipid metabolism was significantly influenced by SHTB administration. Similar to the above results, SHTB increased the concentrations of SCFAs in feces and serum but decreased the concentrations of lipids in serum. Molecular experiments showed that SHTB modulates contents of relevant lipid metabolic enzymes, including CT, ET, PAP, ASMase, SMS, ATGL and HSL in colon tissues. Therefore, the findings demonstrate that SHTB could attenuate intestinal barrier permeability and the inflammatory response by promoting the production of SCFAs and inhibiting lipid accumulation, thereby restoring intestinal flora homeostasis and alleviating STC. These findings offer novel insights into the quality control and therapeutic target of SHTB.

## Supplementary Information

Additional file 1.

## Data Availability

The datasets used and analyzed during the current study are available from the corresponding author upon reasonable request.

## Abbreviations

SHTB: Shouhui Tongbian Capsule
STC: Slow transit constipation
LPS: Lipopolysaccharide
MRW: Maren pills
LC–MS: Liquid chromatography-mass spectrometry
DAO: Diamine oxidase
TNF-α: Tumor necrosis factor alpha
IL-1β: Interleukin 1β
ESI: Electrospray ionization
SPF: Specific pathogen free
MTBE: Methyl-tert-butyl ether
SIM: Selected ion monitoring
TIC: Total ion chromatography
ROI: Regions of interest
PLS-DA: Partial least squares discrimination analysis
BPI: Base peak intensity
SCFAs: Short-chain fatty acids
SMs: Sphingolipids
PLD: Phospholipase
PAP: Phosphatidic acid phosphatase
DGAT: Diacylglycerol acyltransferase
PUFA: Polyunsaturated fatty acids
SMase: Sphingomyelinase
CT: CTP-phosphocholine cytidylyltransferase
ET: CTP-phosphoethanolamine cytidylyltransferase
ASMase: Acid sphingomyelinase
SMS: Sphingomyelin synthase
ATL: Adipose triglyceride lipase
HSL: Hormone-sensitive lipase
MG: Monoglycerides
