# Supplementary material for: Integrated serum pharmacochemistry, 16S rDNA sequencing, and metabolomics to reveal the material basis and mechanism of Shouhui Tongbian capsule against diphenoxylate-induced slow transit constipation in rats
Source: Chin Med. 2024 Oct 11;19:142. doi: 10.1186/s13020-024-01015-8 (PMC11468123; doi:10.1186/s13020-024-01015-8)
Supplement: Supplementary file 1 — Additional file 1. [file 13020_2024_1015_MOESM1_ESM.docx]

Supplementary materials

**Integrated serum pharmacochemistry, 16S rDNA sequencing, and metabolomics to reveal the material basis and mechanism of Shouhui Tongbian capsule against diphenoxylate-induced slow transit constipation in rats**

Jiaying Yang^1,2^, Zhengyu Fang^1^, Jingchun Yao^5^, Pin Zhang^4^, Bojiao Yi^4^, He Xiao^5^, Na Guo^1,3*^, Yongxia Guan^5*^, Guimin Zhang^5*^

^1^ Experimental Research Center, China Academy of Chinese Medical Sciences, Beijing, China

^2^ College of Pharmacy, Heilongjiang University of Traditional Chinese Medicine, Heilongjiang, China

^3^ State Key Laboratory for Quality Ensurance and Sustainable Use of Dao-di Herbs，National Resource Center for Chinese Materia Medica, China Academy of Chinese Medical Sciences，Beijng，100700，P. R. China

^4^ School of Pharmacy, Shenyang Pharmaceutical University, Shenyang 110016, China

^5^ State Key Laboratory of Generic Manufacture Technology of Traditional Chinese Medicine, Lunan Pharmaceutical Group Co. Ltd., Shandong, China

* Correspondence:

Guo Na: guona5246@126.com

Yongxia Guan: 13581069215@163.com;

Guimin Zhang: lunanzhangguiming@yeah.net

# 1. Supplementary Notes

# 1.1 Systematic screening of chemical constituents in SHTB

# The systematic screening of chemical constituents in SHTB was performed using a Waters ACQUITY UPLC system coupled with a Waters Xevo G2XS Q-TOF system (Waters, Milford, MA, USA) equipped with an electrospray ionization (ESI) source conducted in positive and negative ion modes. An ethylene bridged hybrid (BEH) C18 column (2.1 mm × 100 mm, 1.7 μm) was used at 40℃ for chromatographic separation. The mobile phases 0.1% formic acid in water (A) and acetonitrile (B) were used in the following gradient elution profile: 0.0-3.0 min: 95-85% A; 3.0-10.0 min: 85-75% A; 10.0-15.0 min: 75-50% A; 15.0-18.0 min: 50-30% A; 18.0-20.0 min: 30-0% A; 20.0-22.0 min: 0% A and 22.0-24.0 min: 0-95% A. The flow rate was maintained at 0.4 mL/min. The flow rate of desolvation gas was set to 800 L/h at 400 ℃, and the source temperature was maintained at 100 ℃. The capillary and cone voltages were 2000 V and 40 V, respectively. Mass data were acquired in the MSE mode, and the ramp collision energy was set at 10-60 V. Mass data over the range 50-1200 Da were collected using the LockSpray™ interface to ensure accuracy and reproducibility. Masses were calculated in ESI+ and ESI- modes by performing calibration with the reference compound leucine-enkephalin (200 pg/μL), being measured at m/z 556.2771 and 554.2615 for [M+H]^+^ and [M-H]^-^ ions, respectively.

# 1.2 Quantification of main ingredients in SHTB

# The quantification of 21 main ingredients in SHTB were via the Waters ACQUITY UPLC system coupled with a Waters Xevo TQ-S micro system (Waters, Milford, MA, USA) in ESI negative ion mode. The flow rate of the desolvation gas was set to 800 L/h at 400 ◦C, cone gas was at 150 L/h, and capillary voltage and sampling cone voltages were 1.5 kV and 0.4 kV, respectively. Data acquisition was performed in multiple reaction monitoring (MRM) mode. Precursor-to-product ion pairs, optimized cone voltage (CV), and collision energy (CE) were listed in Table S1. The TargetLynx program (v 4.1, Waters, Milford, MA) was used for integrating peak areas, generating calibration curves and calculating concentrations as follows: Sample concentration = sample area* (IS concentration/ IS area).

# To determine the reliability of the test results, the method validation included linearity, repeatability, intra- and interday precisions, stability and recovery test. Linear regression analysis was used to validate the method by the slope, intercept, and correlation coefficient of each calibration curve, and a weighting factor of 1/x was used. For the evaluation of precision, standard solutions were prepared for intra- and inter-precision analyses in three and six separated validation runs, respectively. Recovery was tested by spiking samples with known quantities of standard compounds at 50%, 100%, and 150% concentration ratios, and the recovery rates of 21 main ingredients were calculated by the following formula: Recovery (%) = (Detected amount-Added amount) / Initial amount × 100%.

# The standard calibration curves and linear range of all compounds were shown in Table S2 with satisfactory linearity (r2 > 0.99). 21 main ingredients had a linear range of 0.20 ng/mL to 6400 ng/mL. The LOD ranged from 0.02 - 1500.00 pg/mL and LOQ ranged from 0.20 - 3000.00 pg/mL for all 21 analytes. The intra-day with RSD less than 8.99% and inter-day with RSD less than 9.95% are demonstrated in Table S2. The repeatability was satisfactory with RSD below 9.51%. Stability deviated within 9.57%, indicating that samples were stabled at room temperature for 24 h. Recovery was tested by spiking samples with known quantities of standard compounds at 50%, 100%, and 150% concentration levels (Table S3), low concentration recovery of the 21 compounds was within the range of 94.12-104.80%, medium concentration recovery of the 21 compounds was within the range of 91.68-108.07%, high concentration Recovery of the 21 compounds was within the range of 94.45-109.19%. These results showed no relevant difference in the percent yield recovered using with different concentrations of the compounds. Thus, the 21 analytes can be quantitatively analyzed simultaneously in a relatively short-time using this optimized method. The described UPLC-MS/MS method was subsequently applied to analysis of the SHTB solution.

# 1.3 Targeted metabolomics analysis of lipids and fatty acids in serums

# Lipid and fatty acid separation were achieved using a Waters Acquity UPLC BEH C8 column (2.1 × 100 mm, 1.7 μm; flow rate of 0.26 ml/min). The mobile phases for this separation step were 60% acetonitrile containing 5 mm ammonium formate (A) and 90% isopropanol in acetonitrile containing 5 mm ammonium formate (B). The linear elution gradient settings were as follows: 0-1.0 min, 100% A; 1.0-2.0 min, 100-70% A; 2.0-12.0 min, 70-30% A; 12.0-12.5 min, 30-5% A; 12.5-13.0 min, 5-0% A; 13.0-14.0 min, 0% A; 14.0-14.1 min, 0-100% A; and 14.1-16.0 min, 100% A. The column was warmed to 55°C. A 1-μl injection volume was used in positive ion mode, while a 3μl volume was used in negative ion mode. The details of targeted lipid and fatty acid analyses are discussed in our previously published study [1]. Data acquisition and peak processing were conducted using Skyline (v 2.1) software.

# 1.4 Evaluation of system stability

# Pooled QC samples was prepared for each analysis as described above 2.8 and 2.9. These QC samples were processed for analysis in the sample manner as individual samples. The QC samples were injected every 10 injections and analyzed. This sample was used to validate the stability of the LC-MS and GC-MS system.

# Validation of the LC-MS and GC-MS methods is shown by the repeatability of the spiked internal standards for these QC samples (Tables S7-S8). The low relative standard deviations of peak areas of internal standards indicated that the reproducibility of these measurements over the experimental duration was acceptable. For targeted lipid, fatty acid and SCFAs analyses, the majority of the coefficient of variation values corresponding to the internal standard peak area were < 30%.

# Supplementary Figures and Tables

## Supplementary Figures


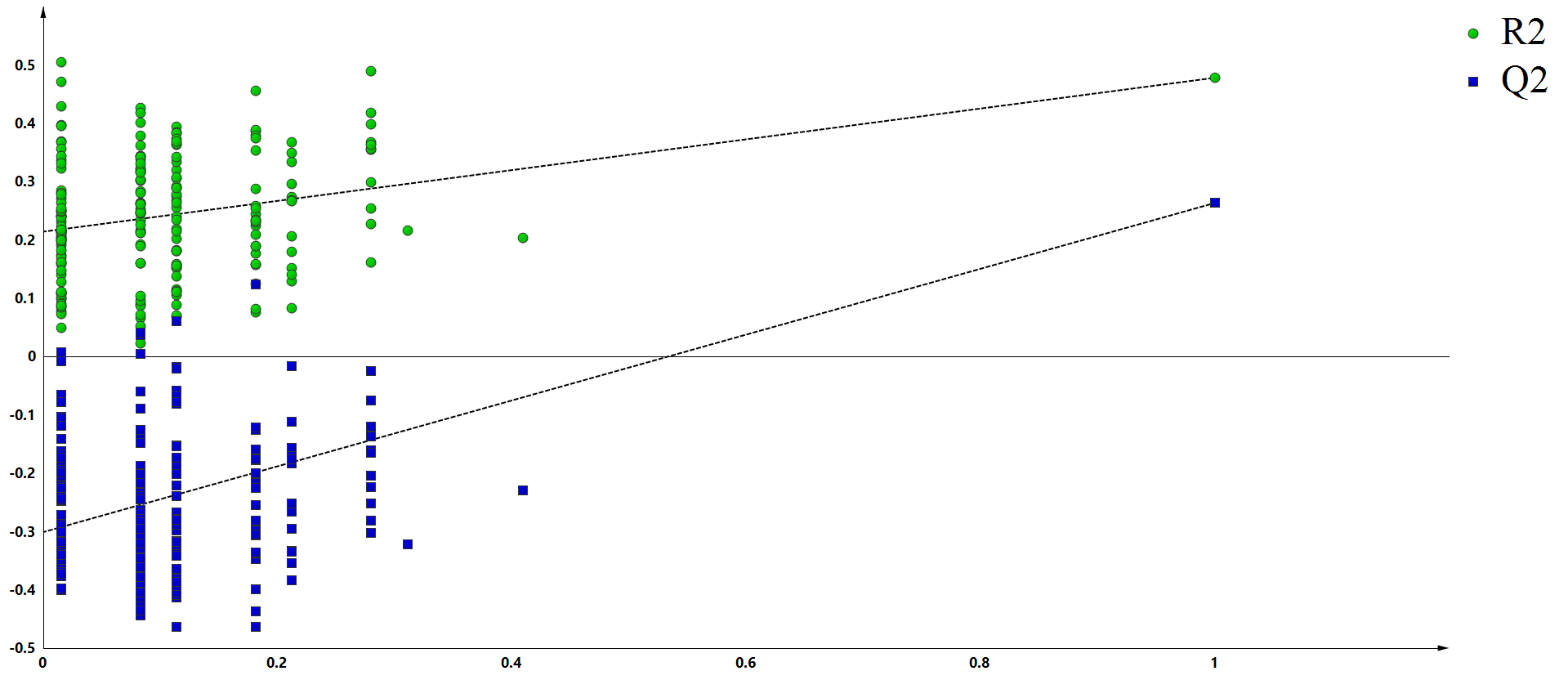


**Figure S1**. Permutation test (n=200) of the PLS-DA model.

## Supplementary Tables

Table S1. MRM collection parameters of 21 main ingredients

| Compound | Parent (m/z) | Daughter (m/z) | CV(V) | CE(V) |
| --- | --- | --- | --- | --- |
| Cassiaside B2 | 918.99 | 270.94 | 90 | 42 |
| Chrysophanol 1-O-β-triglucoside | 738.99 | 252.89 | 14 | 60 |
| Cassiaside C | 594.95 | 270.90 | 52 | 28 |
| Chrysophanol-1-O-β-gentiobioside | 577.00 | 252.95 | 2 | 18 |
| Cassiaside B | 565.00 | 270.94 | 6 | 22 |
| Aurantio-obtusin beta-D-glucoside | 490.90 | 475.93 | 4 | 26 |
| Physcion 8-O-β-glucopyranoside | 480.90 | 282.91 | 12 | 12 |
| Emodin-8-O-β-D-glucopyranoside | 431.07 | 268.89 | 26 | 30 |
| Aloe-emodin-glucoside | 431.01 | 267.91 | 2 | 26 |
| Cassiaside | 419.14 | 257.06 | 4 | 22 |
| Chrysophanol 8-O-glucoside | 414.95 | 252.90 | 28 | 24 |
| Chrysoobtusin | 356.95 | 341.95 | 50 | 20 |
| Obtusin | 342.87 | 327.87 | 2 | 18 |
| Aurantio-obtusin | 328.91 | 313.91 | 8 | 18 |
| 2-hydroxyl emodin-1-methyl ether | 298.9 | 255.88 | 2 | 30 |
| Physcion | 283.10 | 239.90 | 26 | 12 |
| Rhein | 282.94 | 238.87 | 22 | 12 |
| Obtusifolin | 282.91 | 239.76 | 50 | 28 |
| Emodin | 268.89 | 224.90 | 2 | 26 |
| Aloe-emodin | 268.89 | 239.91 | 6 | 22 |
| Chrysophanol | 252.96 | 224.89 | 2 | 26 |

Table S2. Summary of LOD, LOQ, linear, precision, repeatability, and stability of 21 main ingredients in SHTB samples

| Analyte | Calibration curves | r^2^ | Linear range (ng/ml) | LOD (pg/mL) | LOQ (pg/mL) | Precision (%RSD) | | Repeatability (%RSD, n = 6) | Stability (%RSD, n = 6) |
| --- | --- | --- | --- | --- | --- | --- | --- | --- | --- |
|  |  |  |  |  |  | Intra-day (n = 6) | Inter-day (n =3) |  |  |
| Cassiaside B2 | y=5.7261x+142.959 | 0.9987 | 12.50-3200.00 | 400 | 1200.00 | 8.99 | 7.95 | 5.44 | 2.13 |
| Chrysophanol 1-O-β-triglucoside | y=46.225x+1101.81 | 0.9980 | 9.38-2400.00 | 20 | 30.00 | 4.77 | 9.95 | 3.05 | 1.95 |
| Cassiaside C | y=193.586x+4075 | 0.9986 | 9.38-2400.00 | 40 | 70.00 | 2.82 | 7.43 | 2.01 | 0.52 |
| Chrysophanol-1-O-β-gentiobioside | y=214.692x+4499.22 | 0.9916 | 3.91-1000.00 | 20 | 30.00 | 4.26 | 7.06 | 3.01 | 1.16 |
| Cassiaside B | y=700.322x+551.022 | 0.9992 | 3.91-1000.00 | 4 | 7.00 | 2.17 | 7.07 | 1.82 | 1.46 |
| Aurantio-obtusin beta-D-glucoside | y=169.202+5219.12 | 0.9974 | 9.38-2400.00 | 20 | 30.00 | 3.84 | 5.45 | 1.11 | 2.34 |
| physcion 8-O-β-glucopyranoside | y=35.0549x+477.93 | 0.9976 | 4.69-1200.00 | 300 | 600.00 | 3.77 | 9.27 | 3.31 | 4.20 |
| Emodin-8-O-β-D-glucopyranoside | y=1506.74x+19764.6 | 0.9980 | 4.69-1200.00 | 100 | 300.00 | 2.90 | 4.62 | 1.67 | 1.28 |
| Aloe-emodin-glucoside | y=2531.74x+121.02 | 0.9991 | 0.23-60.00 | 10 | 20.00 | 3.36 | 2.53 | 2.07 | 1.99 |
| Cassiaside | y=1626.89x+17517 | 0.9973 | 3.13-800.00 | 20 | 30.00 | 4.56 | 4.23 | 1.66 | 1.41 |
| Chrysophanol 8-O-glucoside | y=220.684x+14.189 | 0.9995 | 0.31-80.00 | 150 | 300.00 | 4.09 | 6.04 | 3.71 | 3.10 |
| Chrysoobtusin | y=158.614x-2564.78 | 0.9956 | 3.91-1000.00 | 60 | 110.00 | 6.50 | 8.18 | 3.43 | 3.39 |
| Obtusin | y=528.304x+972.456 | 0.9995 | 1.76-450.00 | 60 | 600.00 | 5.54 | 5.56 | 2.80 | 3.65 |
| Aurantio-obtusin | y=820.252x+4769.94 | 0.9989 | 3.13-800.00 | 20 | 60.00 | 3.19 | 7.03 | 3.02 | 3.29 |
| 2-hydroxyl emodin-1-methyl ether | y=2769.16x+1542.08 | 0.9987 | 0.31-80.00 | 0.02 | 0.20 | 4.18 | 4.83 | 1.99 | 1.81 |
| Physcion | y=19.4411x-407.281 | 0.9911 | 3.13-800.00 | 1500 | 3000.00 | 4.84 | 2.24 | 9.51 | 9.57 |
| Rhein | y=1699.74x+509.026 | 0.9996 | 0.31-80.00 | 150 | 300.00 | 4.00 | 4.04 | 6.59 | 2.41 |
| Obtusifolin | y=647.606x+1557.4 | 0.9992 | 1.56-400.00 | 70 | 150.00 | 8.88 | 9.67 | 2.50 | 2.89 |
| Emodin | y=1073.37x+8286.58 | 0.9940 | 1.56-400.00 | 6 | 10.00 | 7.51 | 5.95 | 2.47 | 1.49 |
| Aloe-emodin | y=177.104x+2523.91 | 0.9995 | 25.00-6400.00 | 2 | 3.00 | 7.09 | 2.50 | 6.10 | 1.02 |
| Chrysophanol | y=825.252x+193.28 | 0.9997 | 0.20-50.00 | 100 | 200.00 | 7.50 | 7.03 | 3.91 | 4.66 |

Table S3. Recovery for 21 main ingredients at low, medium, and high concentrations (n = 3)

| Analyte | Initial amoμnt (μg) | Low concentration (50%) | | | | Mediμm concentration (100%) | | | | High concentration (150%) | | | |
| --- | --- | --- | --- | --- | --- | --- | --- | --- | --- | --- | --- | --- | --- |
|  |  | Added amoμnt (μg) | Detected amoμnt (μg) | Recovery | | Added amoμnt (μg) | Detected amoμnt (μg) | Recovery | | Added amoμnt (μg) | Detected amoμnt (μg) | Recovery | |
|  |  |  |  | (%, mean ± SD) | RSD% |  |  | (%, mean ± SD) | RSD% |  |  | (%, mean ± SD) | RSD% |
| Cassiaside B2 | 17.35 | 8.00 | 25.63 | 101.59±4.46 | 4.39 | 16.00 | 33.37 | 100.08±4.78 | 4.78 | 24.00 | 40.74 | 96.49±9.77 | 10.12 |
| Chrysophanol 1-O-β-triglucoside | 25.95 | 12.60 | 39.43 | 103.4±7.46 | 7.21 | 25.20 | 52.28 | 104.37±3.08 | 2.95 | 37.80 | 63.33 | 98.39±2.61 | 2.66 |
| Cassiaside C | 66.04 | 30.00 | 92.74 | 95.01±2.01 | 2.11 | 60.00 | 126.28 | 100.37±4.25 | 4.24 | 90.00 | 155.95 | 99.87±3.01 | 3.02 |
| Chrysophanol-1-O-β-gentiobioside | 12.53 | 6.25 | 19.00 | 101.73±6.99 | 6.88 | 12.50 | 25.17 | 101.14±4.02 | 3.97 | 18.75 | 31.95 | 105.37±2.10 | 1.99 |
| Cassiaside B | 7.09 | 3.40 | 10.72 | 103.29±2.61 | 2.52 | 6.80 | 14.02 | 101.88±5.66 | 5.56 | 10.20 | 17.71 | 105.98±4.40 | 4.15 |
| Aurantio-obtusin beta-D-glucoside | 21.22 | 10.00 | 31.34 | 100.58±8.88 | 8.82 | 20.00 | 41.75 | 102.51±5.46 | 5.32 | 30.00 | 50.93 | 98.67±4.17 | 4.22 |
| physcion 8-O-β-glucopyranoside | 7.60 | 3.80 | 10.95 | 94.12±6.20 | 6.59 | 7.60 | 15.56 | 104.76±7.13 | 6.80 | 11.40 | 19.63 | 108.34±2.48 | 2.29 |
| Emodin-8-O-β-D-glucopyranoside | 25.32 | 12.00 | 36.49 | 96.70±1.07 | 1.10 | 24.00 | 50.26 | 103.71±2.83 | 2.73 | 36.00 | 61.37 | 100.17±0.58 | 0.58 |
| Aloe-emodin-glucoside | 0.21 | 0.10 | 0.30 | 99.51±3.90 | 3.92 | 0.20 | 0.41 | 101.62±8.6 | 8.46 | 0.30 | 0.50 | 95.14±3.65 | 3.84 |
| Cassiaside | 19.23 | 9.50 | 27.52 | 93.74±4.70 | 5.01 | 19.00 | 38.25 | 100.14±3.54 | 3.54 | 28.50 | 48.23 | 102.63±1.62 | 1.58 |
| Chrysophanol 8-O-glucoside | 0.10 | 0.05 | 0.14 | 101.05±4.21 | 4.17 | 0.09 | 0.19 | 108.07±5.19 | 4.80 | 0.14 | 0.23 | 98.25±6.43 | 6.55 |
| Chrysoobtusin | 4.58 | 2.25 | 6.71 | 97.33±2.75 | 2.83 | 4.50 | 8.70 | 91.68±1.42 | 1.55 | 6.75 | 10.97 | 92.16±3.43 | 3.72 |
| Obtusin | 3.44 | 1.70 | 5.10 | 99.08±6.11 | 6.17 | 3.40 | 6.84 | 100.18±2.53 | 2.53 | 5.10 | 8.76 | 106.61±4.32 | 4.06 |
| Aurantio-obtusin | 15.15 | 7.50 | 22.89 | 101.58±1.53 | 1.51 | 15.00 | 30.84 | 104.56±4.67 | 4.47 | 22.50 | 38.93 | 108.47±0.68 | 0.63 |
| 2-hydroxyl emodin-1-methyl ether | 0.50 | 0.25 | 0.77 | 103.82±3.49 | 3.36 | 0.50 | 1.03 | 106.17±2.07 | 1.95 | 0.75 | 1.28 | 106.37±2.91 | 2.74 |
| Physcion | 3.11 | 1.60 | 4.55 | 95.08±2.24 | 2.35 | 3.20 | 6.26 | 98.35±3.42 | 3.48 | 4.80 | 7.74 | 94.45±1.92 | 2.04 |
| Rhein | 0.56 | 0.28 | 0.87 | 104.80±5.21 | 4.97 | 0.56 | 1.13 | 102.13±2.4 | 2.35 | 0.84 | 1.40 | 99.59±7.75 | 7.78 |
| Obtusifolin | 4.18 | 2.00 | 6.11 | 98.23±4.59 | 4.68 | 4.00 | 8.07 | 97.35±0.98 | 1.01 | 6.00 | 10.36 | 104.27±2.72 | 2.61 |
| Emodin | 7.59 | 3.70 | 11.41 | 101.52±6.38 | 6.28 | 7.40 | 15.08 | 101.21±3.82 | 3.77 | 11.10 | 18.87 | 102.37±3.05 | 2.97 |
| Aloe-emodin | 151.69 | 76.00 | 220.63 | 95.34±2.81 | 2.95 | 152.00 | 291.27 | 91.81±0.82 | 0.89 | 228.00 | 373.99 | 96.24±1.54 | 1.60 |
| Chrysophanol | 0.06 | 0.03 | 0.09 | 95.68±4.29 | 4.48 | 0.06 | 0.11 | 99.46±7.31 | 7.35 | 0.09 | 0.16 | 109.19±0.94 | 0.86 |

Table S4. Analysis of chemical constituents of Shouhui Tongbian capsule based on UPLC-Q-TOF-MS

| Number | R_T_ (min) | Ion mode | Assigned name | Molecular formula | Theoretical extract mass (Da) | Mean measured mass (Da) | Error (ppm) | Fragmetation |
| --- | --- | --- | --- | --- | --- | --- | --- | --- |
| 1 | 0.55 | ESI- | Tyrosine* | C_9_H_11_NO_3_ | 180.0661 | 180.0655 | -3.33 | - |
| 2 | 0.55 | ESI- | Arginine* | C_6_H_14_N_4_O_2_ | 173.1039 | 173.104 | 0.58 | - |
| 3 | 0.56 | ESI- | Aspartic acid* | C_4_H_7_NO_4_ | 132.0297 | 132.0297 | 0.00 | - |
| 4 | 0.69 | ESI- | trans-4-Hydroxy-L-Proline* | C_5_H_9_NO_3_ | 130.0504 | 130.0509 | 3.84 | - |
| 5 | 0.69 | ESI- | Isoleucine* | C_6_H_13_NO_2_ | 130.0868 | 130.0862 | -4.61 | - |
| 6 | 0.69 | ESI- | Leucine* | C_6_H_13_NO_2_ | 130.0868 | 130.0862 | -4.61 | - |
| 7 | 0.7 | ESI+ | Gallic acid* | C_7_H_6_O_5_ | 171.0293 | 171.0284 | -5.26 | 153.02，126.96，109.01 |
| 8 | 1.31 | ESI- | Phenylalanine* | C_9_H_11_NO_2_ | 164.0712 | 164.0711 | -0.61 | - |
| 9 | 1.61 | ESI- | 3,4-Dihydroxybenzoic acid | C_7_H_6_O_4_ | 153.0188 | 153.018 | -5.23 | 137.03，93.04 |
| 10 | 2.02 | ESI- | Procyanidin B1 | C_30_H_26_O_12_ | 577.1424 | 577.1395 | -5.02 | 451.13，425.04，289.07 |
| 11 | 2.02 | ESI- | Procyanidin B2 | C_30_H_26_O_12_ | 577.1424 | 577.1395 | -5.02 | 451.13，425.04，289.07 |
| 12 | 2.02 | ESI- | Tryptophan* | C_11_H_12_N_2_O_2_ | 203.0821 | 203.082 | -0.49 | - |
| 13 | 2.33 | ESI- | (-)-Epicatechin | C_15_H_14_O_6_ | 289.0712 | 289.0716 | 1.38 | 245.08，205.05，123.05 |
| 14 | 2.41 | ESI- | 5-Methylfurfural | C_6_H_6_O_2_ | 109.029 | 109.0288 | -1.83 | - |
| 15 | 2.44 | ESI- | AloESIin* | C_19_H_22_O_9_ | 393.1186 | 393.1198 | 3.05 | 273.08，245.08 |
| 16 | 2.46 | ESI+ | Catechin* | C_15_H_14_O_6_ | 291.0869 | 291.0861 | -2.75 | 138.97，122.97，164.96 |
| 17 | 2.46 | ESI+ | 4-Hydroxybenzoic acid* | C_7_H_6_O_3_ | 139.0395 | 139.0399 | 2.88 | 95.0499 |
| 18 | 2.46 | ESI- | 3'-hydroxy Puerarin* | C_21_H_20_O_10_ | 433.1135 | 433.111 | -5.77 | 283.06，267.07 |
| 19 | 2.54 | ESI+ | Chlorogenic acid* | C_16_H_18_O_9_ | 355.1029 | 355.103 | 0.28 | 163.04，89.04， |
| 20 | 2.85 | ESI- | Kynurenic acid* | C_10_H_12_N_2_O_3_ | 207.077 | 207.0769 | -0.48 | - |
| 21 | 3.08 | ESI+ | puerarin* | C_21_H_20_O_9_ | 417.1186 | 417.1191 | 1.20 | - |
| 22 | 3.15 | ESI+ | vanillic acid* | C_8_H_8_O_4_ | 169.0501 | 169.05005 | -0.30 | 124.96 |
| 23 | 3.25 | ESI+ | Puerarin 6''-O-xyloside* | C_26_H_28_O_13_ | 549.1608 | 549.1599 | -1.64 | - |
| 24 | 3.26 | ESI+ | 3’-Methoxy Puerarin* | C_22_H_22_O_10_ | 447.1291 | 447.13 | 2.01 | - |
| 25 | 3.46 | ESI- | Ornithine* | C_5_H_12_N_2_O_2_ | 131.0821 | 131.0827 | 4.58 | - |
| 26 | 3.52 | ESI- | Asparaginate* | C_4_H_8_N_2_O_3_ | 131.0457 | 131.0447 | -7.63 | - |
| 27 | 3.96 | ESI+ | Glycitin* | C_22_H_22_O_10_ | 447.1291 | 447.1277 | -3.13 | 285.08，270.05，242.06 |
| 28 | 4.08 | ESI- | (+)-Gallocatechin | C_15_H_14_O_7_ | 305.0661 | 305.065 | -3.61 | 137.03，109.03，125.03 |
| 29 | 4.13 | ESI+ | Daidzin* | C_21_H_20_O_9_ | 417.1186 | 417.1185 | -0.24 | 227.07，137.03 |
| 30 | 4.14 | ESI- | Cinnamic acid | C_9_H_8_O_2_ | 147.0446 | 147.0439 | -4.76 | 103.06，77.04 |
| 31 | 4.14 | ESI- | 3',4',7-Trihydroxyflavone | C_15_H_10_O_5_ | 269.045 | 269.0459 | 3.35 | 224.05，197.06 |
| 32 | 4.18 | ESI- | Epigallocatechin | C_15_H_14_O_7_ | 305.0661 | 305.0663 | 0.66 | 137.03，109.03，125.03 |
| 33 | 4.26 | ESI+ | Vitexin* | C_21_H_20_O_10_ | 433.1135 | 433.1132 | -0.69 | 313.08，283.07 |
| 34 | 4.44 | ESI+ | trans-Ferulic acid(4-Hydroxy-3-methoxycinnamicacid)* | C_10_H_10_O_4_ | 195.0657 | 195.0666 | 4.61 | - |
| 35 | 4.49 | ESI- | Aloe-emodin-8-O-beta-D-glucopyranoside* | C_21_H_20_O_10_ | 431.0978 | 431.1003 | 5.80 | 431.09 |
| 36 | 4.49 | ESI+ | Ferulic acid* | C_10_H_10_O_4_ | 195.0657 | 195.0676 | 9.74 | 177.06，145.03，89.04 |
| 37 | 4.49 | ESI- | (-)-Catechin gallate | C_22_H_18_O_10_ | 441.0822 | 441.0836 | 3.17 | 169.02，289.07，125.02 |
| 38 | 4.49 | ESI- | (-)-Epicatechin gallate | C_22_H_18_O_10_ | 441.0822 | 441.0836 | 3.17 | 169.02，289.07，125.02 |
| 39 | 4.57 | ESI+ | p-Hydroxybenzoic acid | C_7_H_6_O_3_ | 139.0395 | 139.0389 | -4.32 | 121.03，95.05 |
| 40 | 4.57 | ESI- | 2,3,5,4'-Tetrahydroxystilbene 2-O-glucoside | C_20_H_22_O_9_ | 405.1186 | 405.1185 | -0.25 | 243.07 |
| 41 | 4.57 | ESI- | OxyrESIveratrol 3'-O-beta-D-glucopyranoside | C_20_H_22_O_9_ | 405.1186 | 405.1185 | -0.25 | 397.11，243.07，225.06 |
| 42 | 4.57 | ESI- | Rhombifoline | C_15_H_20_N_2_O | 243.1497 | 243.1485 | -4.94 | - |
| 43 | 4.62 | ESI+ | Isovitexin* | C_21_H_20_O_10_ | 433.1135 | 433.1164 | 6.70 | 313.08，283.07 |
| 44 | 4.75 | ESI- | Hyperoside | C_21_H_20_O_12_ | 463.0877 | 463.0859 | -3.89 | 299.09，271.06 |
| 45 | 4.8 | ESI+ | Tectorigenin 7-O-xylosylglucoside* | C_27_H_30_O_15_ | 595.1663 | 595.1664 | 0.17 | 301.07，286.05，317.06 |
| 46 | 4.8 | ESI- | 3',4'-Dihydroxyflavone | C_15_H_10_O_4_ | 253.0501 | 253.0499 | -0.79 | 133.03，132.02 |
| 47 | 4.93 | ESI+ | Ellagic acid* | C_14_H_6_O_8_ | 325.009 | 325.008 | -3.08 | 135.05，93.01 |
| 48 | 4.93 | ESI+ | 4''-methyloxy-Daidzin* | C_22_H_22_O_9_ | 431.1342 | 431.1331 | -2.55 | 255.07，199.08，137.02 |
| 49 | 4.93 | ESI- | Eriocitrin | C_27_H_32_O_15_ | 595.1663 | 595.1665 | 0.34 | - |
| 50 | 5.01 | ESI+ | isoquercetin* | C_21_H_20_O_12_ | 465.1033 | 465.1012 | -4.52 | 303.04， 229.05 |
| 51 | 5.01 | ESI+ | Genistin* | C_21_H_20_O_10_ | 433.1135 | 433.1166 | 7.16 | - |
| 52 | 5.01 | ESI+ | genistein-8-c-glucoside* | C_21_H_20_O_10_ | 433.1135 | 433.112 | -3.46 | 313.07，283.06，415.10 |
| 53 | 5.19 | ESI+ | Kaempferol 3-rutinoside* | C_27_H_30_O_15_ | 595.1663 | 595.1682 | 3.19 | 287.07，331.00，448.99 |
| 54 | 5.32 | ESI+ | kaempferitrin* | C_27_H_30_O_14_ | 579.1714 | 579.1733 | 3.28 | - |
| 55 | 5.37 | ESI+ | tectoridin* | C_22_H_22_O_11_ | 463.124 | 463.1271 | 6.69 | 301.07，286.04 |
| 56 | 5.55 | ESI+ | 6''-O-Malonyldaidzin* | C_24_H_22_O_12_ | 503.119 | 503.1198 | 1.59 | - |
| 57 | 5.67 | ESI- | 3',4'-Dimethoxyflavone | C_17_H_14_O_4_ | 281.0814 | 281.0833 | 6.76 | 131.04 |
| 58 | 5.67 | ESI- | Narirutin | C_27_H_32_O_14_ | 579.1714 | 579.1713 | -0.17 | - |
| 59 | 5.7 | ESI+ | 4-Methylumbelliferone* | C_10_H_8_O_3_ | 177.0552 | 177.0554 | 1.13 | 105.07，77.04，105.07 |
| 60 | 5.93 | ESI+ | Salicylic acid* | C_7_H_6_O_3_ | 139.0395 | 139.0391 | -2.88 | 121.03，93.06 |
| 61 | 5.93 | ESI+ | Rhapontin | C_21_H_24_O_9_ | 421.1499 | 421.1489 | -2.37 | - |
| 62 | 6.12 | ESI- | Aloenin | C_19_H_22_O_10_ | 409.1135 | 409.1157 | 5.38 | 215.04，171.05，188.05 |
| 63 | 6.16 | ESI+ | 3',4',7-Trihydroxyisoflavone* | C_15_H_10_O_5_ | 271.0606 | 271.0598 | -2.95 | 137.02，225.06，215.07 |
| 64 | 6.47 | ESI+ | scoparone* | C_11_H_10_O_4_ | 207.0657 | 207.0663 | 2.90 | 151.08，191.03，107.05 |
| 65 | 6.47 | ESI+ | 4''-methyloxy-Genistin* | C_22_H_22_O_10_ | 447.1291 | 447.1305 | 3.13 | 271.06，293.03，153.02 |
| 66 | 6.47 | ESI+ | 4'-methoxypuerarin* | C_22_H_22_O_10_ | 447.1291 | 447.1305 | 3.13 | - |
| 67 | 6.61 | ESI- | Naringin* | C_27_H_32_O_14_ | 579.1714 | 579.1714 | 0.00 | 271.06，151.00 |
| 68 | 6.73 | ESI- | Cassiaside B2* | C_39_H_52_O_25_ | 919.2719 | 919.2776 | 6.20 | 271.06，255.07，647.20 |
| 69 | 6.86 | ESI- | Diosmin | C_28_H_32_O_15_ | 607.1663 | 607.1666 | 0.49 | - |
| 70 | 6.97 | ESI+ | 6'-O-Malonylgenistin* | C_24_H_22_O_13_ | 517.0951 | 517.0911 | -7.74 | - |
| 71 | 7.04 | ESI- | Cassiaside * | C_20_H_20_O_10_ | 419.0978 | 419.0983 | 1.19 | 257.04，213.05 |
| 72 | 7.12 | ESI+ | Corchoionoside C((6S,9S)-Roseoside)* | C_19_H_30_O_8_ | 387.2019 | 387.202 | 0.26 | 203.05，185.05，247.05 |
| 73 | 7.22 | ESI- | Aloins* | C_21_H_22_O_9_ | 417.1186 | 417.1201 | 3.60 | 297.08，268.07 |
| 74 | 7.35 | ESI- | Chrysophanol 1-O-β-triglucoside* | C_33_H_40_O_19_ | 739.2086 | 739.2104 | 2.44 | 253.05 |
| 75 | 7.35 | ESI- | Aurantio-obtusin beta-D-glucoside* | C_23_H_24_O_12_ | 491.119 | 491.1193 | 0.61 | 242.02，461.07，476.09 |
| 76 | 7.35 | ESI- | Notoginsenoside Fa | C_59_H_100_O_27_ | 1239.6374 | 1239.636 | -1.13 | - |
| 77 | 7.48 | ESI+ | Puerol B* | C_24_H_26_O_10_ | 475.1604 | 475.1634 | 6.31 | - |
| 78 | 7.53 | ESI- | AloerESIin D* | C_29_H_32_O_11_ | 555.1866 | 555.1874 | 1.44 | 145.03，117.04 |
| 79 | 7.53 | ESI+ | Nomilin | C_28_H_34_O_9_ | 560.2258 | 560.2218 | -7.14 | - |
| 80 | 7.66 | ESI- | 7-O-Methylaloeresin A | C_29_H_30_O_11_ | 553.171 | 553.1714 | 0.72 | - |
| 81 | 7.79 | ESI- | Chrysophanol-1-O-β-gentiobioside* | C_27_H_30_O_14_ | 577.1557 | 577.1578 | 3.64 | 253.05，225.05 |
| 82 | 7.79 | ESI+ | Liquiritigenin* | C_15_H_12_O_4_ | 257.0814 | 257.0809 | -1.94 | 136.96，147.05，80.97 |
| 83 | 7.79 | ESI+ | Panasenoside | C_27_H_30_O_16_ | 633.15 | 633.149 | -1.58 | 287.07 |
| 84 | 7.84 | ESI- | Aloin A* | C_21_H_22_O_9_ | 417.1186 | 417.1209 | 5.51 | 297.08，268.07 |
| 85 | 7.89 | ESI- | Polydatin | C_20_H_22_O_8_ | 389.1236 | 389.1256 | 5.14 | 185.06，227.07 |
| 86 | 7.89 | ESI- | Rubrofusarin triglucoside | C_33_H_42_O_20_ | 757.2191 | 757.2228 | 4.89 | 258.05，230.06 |
| 87 | 7.89 | ESI- | 8-Hydroxy-3,5,6,7,3´,4´-hexamethoxyflavone | C_21_H_22_O_9_ | 417.1186 | 417.1204 | 4.32 | - |
| 88 | 8.02 | ESI+ | Daidzein* | C_15_H_10_O_4_ | 255.0657 | 255.0659 | 0.78 | 199.08 |
| 89 | 8.02 | ESI+ | Ononin* | C_22_H_22_O_9_ | 431.1342 | 431.1373 | 7.19 | - |
| 90 | 8.1 | ESI- | Hesperidin | C_28_H_34_O_15_ | 609.1819 | 609.1816 | -0.49 | 301.07，286.05 |
| 91 | 8.2 | ESI+ | 7-Methoxycoumarin* | C_10_H_8_O_3_ | 177.0552 | 177.055 | -1.13 | 121.07，133.07，77.04 |
| 92 | 8.33 | ESI- | Cassiaside C* | C_27_H_32_O_15_ | 595.1663 | 595.166 | -0.50 | 270.91，255.91 |
| 93 | 8.33 | ESI- | Rubrofusarin gentiobioside | C_27_H_32_O_15_ | 595.1663 | 595.1672 | 1.51 | 258.05，230.06 |
| 94 | 8.33 | ESI- | Isorubrofusarin-6-O-β-gentiobioside | C_27_H_32_O_15_ | 595.1663 | 595.1671 | 1.34 | 258.05，230.06 |
| 95 | 8.46 | ESI+ | Glycitein* | C_16_H_12_O_5_ | 285.0763 | 285.0754 | -3.16 | 270.05，242.06，118.04 |
| 96 | 8.48 | ESI- | Thermopsoside | C_22_H_22_O_11_ | 461.1084 | 461.1068 | -3.47 | 283.02，255.03 |
| 97 | 8.59 | ESI+ | notoginsenoside FP1 | C_47_H_80_O_18_ | 955.5291 | 955.5313 | 2.30 | 781.4，751.43，619.4 |
| 98 | 8.71 | ESI- | Trans-resveratrol 4'-O-β-D-glucuronic acid | C_20_H_20_O_9_ | 403.1029 | 403.1053 | 5.95 | 389.04，377.12.359.11 |
| 99 | 8.77 | ESI+ | luteolin* | C_15_H_10_O_6_ | 287.0556 | 287.0576 | 6.97 | 153.02，135.04，89.04 |
| 100 | 8.89 | ESI+ | Auraptene | C_19_H_22_O_3_ | 344.1624 | 344.1597 | -7.85 | - |
| 101 | 8.9 | ESI+ | Quercetin* | C_15_H_10_O_7_ | 303.0505 | 303.0503 | -0.66 | 109.03 |
| 102 | 8.95 | ESI+ | Notoginsenoside R2 | C_41_H_70_O_13_ | 771.4894 | 771.4908 | 1.81 | 753.48，621.47 |
| 103 | 9.08 | ESI- | Cassiaside B* | C_26_H_30_O_14_ | 565.1557 | 565.1577 | 3.54 | 271.06，256.03，228.04 |
| 104 | 9.13 | ESI+ | 3'-Methoxydaidzein* | C_16_H_12_O_5_ | 285.0763 | 285.075 | -4.56 | 270.05，213.06，137.02 |
| 105 | 9.13 | ESI+ | Calycosin* | C_16_H_12_O_5_ | 285.0763 | 285.075 | -4.56 | 270.05，213.06，137.02 |
| 106 | 9.21 | ESI- | Neohesperidin | C_28_H_34_O_16_ | 609.1819 | 609.1816 | -0.49 | 301.07 |
| 107 | 9.38 | ESI- | 20-Gluco-ginsenoside Rf | C_48_H_82_O_19_ | 961.5372 | 961.5405 | 3.43 | 961.06，637.01 |
| 108 | 9.39 | ESI+ | Ginsenoside Rg1* | C_42_H_72_O_14_ | 801.5 | 801.5015 | 1.87 | 639.45 |
| 109 | 9.39 | ESI+ | ginsenoside La | C_42_H_70_O_13_ | 783.4895 | 783.4892 | -0.38 | 621.44，603.43，457.37 |
| 110 | 9.43 | ESI+ | Ginsenoside Re* | C_48_H_82_O_18_ | 947.5579 | 947.5606 | 2.85 | 785.51，639.44，477.37 |
| 111 | 10.18 | ESI+ | Ginsenoside Rf* | C_42_H_72_O_14_ | 801.5 | 801.5023 | 2.87 | 639.45 |
| 112 | 10.18 | ESI+ | malonyl-ginsenoside Rg1 | C_45_H_74_O_17_ | 909.4815 | 909.4815 | 0.00 | 477.38 |
| 113 | 10.31 | ESI+ | Kakkalide* | C_28_H_32_O_15_ | 609.1819 | 609.1824 | 0.82 | - |
| 114 | 10.36 | ESI- | Emodin-8-O-β-D-glucopyranoside* | C_21_H_20_O_10_ | 431.0978 | 431.0981 | 0.70 | 269.05，225.06，241.05 |
| 115 | 10.44 | ESI- | Toralactone | C_15_H_12_O_5_ | 271.0606 | 271.0605 | -0.37 | 241.05，253.05 |
| 116 | 10.44 | ESI- | Poncirin | C_28_H_34_O_14_ | 593.187 | 593.1873 | 0.51 | 285.08，164.01，151.00 |
| 117 | 10.49 | ESI- | Chrysophanol 8-O-glucoside* | C_21_H_20_O_9_ | 415.1029 | 415.1018 | -2.65 | 253.05 |
| 118 | 10.49 | ESI- | Aloe-emodin-glucoside* | C_21_H_20_O_10_ | 431.0978 | 431.0962 | -3.71 | 268.07 |
| 119 | 10.57 | ESI+ | Genistein* | C_15_H_10_O_5_ | 271.0606 | 271.0597 | -3.32 | 253.05，241.05 |
| 120 | 10.62 | ESI- | Chrysophanol* | C_15_H_10_O_4_ | 253.0501 | 253.0506 | 1.98 | 225.05，181.88 |
| 121 | 10.62 | ESI+ | Isosakuranetin | C_16_H_14_O_5_ | 287.0919 | 287.0912 | -2.44 | 161.06，153.01 |
| 122 | 10.75 | ESI+ | Coumestrol* | C_15_H_8_O_5_ | 269.045 | 269.046 | 3.72 | 211.04，239.03，415.04 |
| 123 | 11.1 | ESI+ | Tectorigenin* | C_16_H_12_O_6_ | 301.0712 | 301.0723 | 3.65 | 286.05，168.01，69.07 |
| 124 | 11.24 | ESI+ | Kaempferol* | C_15_H_10_O_6_ | 287.0556 | 287.0548 | -2.79 | 153.03，121.04 |
| 125 | 11.3 | ESI+ | Isorhamnetin* | C_16_H_12_O_7_ | 317.0661 | 317.0646 | -4.73 | 153.02 |
| 126 | 11.41 | ESI- | Hesperetin | C_16_H_14_O_6_ | 301.0712 | 301.071 | -0.66 | - |
| 127 | 11.44 | ESI+ | Notoginsenoside R4 | C_59_H_100_O_27_ | 1241.653 | 1241.6526 | -0.32 | 899.5 |
| 128 | 11.81 | ESI- | Nobiletin | C_21_H_22_O_8_ | 401.1236 | 401.1236 | 0.00 | 375.11，343.08 |
| 129 | 12.16 | ESI- | physcion 8-O-β-glucopyranoside* | C_22_H_22_O_10_ | 445.1135 | 445.115 | 3.37 | 283.06 |
| 130 | 12.42 | ESI+ | Pseudo-ginsenoside RT5* | C_36_H_62_O_10_ | 655.4421 | 655.4423 | 0.31 | - |
| 131 | 12.42 | ESI- | RESIveratrol | C_14_H_12_O_3_ | 227.0708 | 227.071 | 0.88 | 185.06，143.04 |
| 132 | 12.52 | ESI+ | Isoliquiritigenin* | C_15_H_12_O_4_ | 257.0814 | 257.0828 | 5.45 | - |
| 133 | 12.52 | ESI- | Pseudoginsenoside F11* | C_42_H_72_O_14_ | 799.4844 | 799.4824 | -2.50 | 799.48，653.42，491.37 |
| 134 | 12.73 | ESI- | 2-hydroxyl emodin-1-methyl ether* | C_16_H_12_O_6_ | 299.0556 | 299.0564 | 2.68 | 256.04，284.03，226.99 |
| 135 | 12.73 | ESI+ | Formononetin* | C_16_H_12_O_4_ | 269.0814 | 269.0822 | 2.97 | - |
| 136 | 12.73 | ESI+ | Icaritin | C_21_H_20_O_6_ | 369.1388 | 369.1371 | -4.61 | 147.04 |
| 137 | 12.78 | ESI+ | notoginsenoside O | C_52_H_88_O_21_ | 1049.5897 | 1049.5887 | -0.95 | 780.54，737.48，621.44 |
| 138 | 12.78 | ESI+ | notoginsenoside P | C_52_H_88_O_21_ | 1049.5897 | 1049.5887 | -0.95 | 780.54，737.48，621.44 |
| 139 | 12.83 | ESI- | Aurantio-obtusin* | C_17_H_14_O_7_ | 329.0661 | 329.0665 | 1.22 | 314.04，299.02，271.02 |
| 140 | 12.83 | ESI+ | notoginsenoside L | C_53_H_90_O_22_ | 1079.6003 | 1079.5978 | -2.32 | 1061.58，780.52，737.48 |
| 141 | 12.83 | ESI+ | Ginsenoside-Ra0 | C_60_H_102_O_28_ | 1271.6636 | 1271.6692 | 4.40 | 1253.64，929.5552 |
| 142 | 12.83 | ESI- | Tricin | C_17_H_14_O_7_ | 329.0661 | 329.066 | -0.30 | 271.03，243.03，203.03 |
| 143 | 12.96 | ESI+ | Ginsenoside Ra3* | C_59_H_100_O_27_ | 1241.653 | 1241.6552 | 1.77 | 947.56，785.51，623.44 |
| 144 | 12.96 | ESI+ | Ginsenoside Rb1* | C_54_H_92_O_23_ | 1109.6108 | 1109.6116 | 0.72 | 947.56，785.51，623.44 |
| 145 | 12.96 | ESI+ | Ginsenoside Rg2* | C_42_H_72_O_13_ | 785.5051 | 785.5057 | 0.76 | 639.45，393.3514 |
| 146 | 12.96 | ESI+ | gypenoside XVII | C_48_H_82_O_18_ | 947.558 | 947.5598 | 1.90 | 785.51，767.50，605.44 |
| 147 | 13.04 | ESI+ | Ginsenoside F3* | C_41_H_70_O_13_ | 771.4895 | 771.4937 | 5.44 | 447.36，259.08，131.05 |
| 148 | 13.04 | ESI+ | Ginsenoside F5* | C_41_H_70_O_13_ | 771.4895 | 771.4937 | 5.44 | 639.45，477.37 |
| 149 | 13.04 | ESI+ | malonyl-ginsenoside Rb1 | C_57_H_94_O_26_ | 1195.6112 | 1195.6112 | 0.00 | 1109.05，1015.02，785.51 |
| 150 | 13.04 | ESI- | Torachrysone | C_14_H_14_O_4_ | 245.0814 | 245.0813 | -0.41 | 203.07，213.06，229.05 |
| 151 | 13.14 | ESI- | Aloe-emodin* | C_15_H_10_O_5_ | 269.045 | 269.045 | 0.00 | 116.92，107.05 |
| 152 | 13.14 | ESI+ | Ginsenoside F4* | C_42_H_70_O_12_ | 767.4946 | 767.4947 | 0.13 | 623.35，459.38 |
| 153 | 13.14 | ESI+ | Ginsenoside Ra1* | C_58_H_98_O_26_ | 1211.6425 | 1211.6415 | -0.83 | 789.48，623.44 |
| 154 | 13.14 | ESI+ | Ginsenoside Ra2* | C_58_H_98_O_26_ | 1211.6425 | 1211.641 | -1.24 | 785.51，623.44， |
| 155 | 13.14 | ESI+ | Ginsenoside Rc* | C_53_H_90_O_22_ | 1079.6002 | 1079.5992 | -0.93 | 407.37，425.38，325.11 |
| 156 | 13.22 | ESI+ | irisolidone* | C_17_H_14_O_6_ | 315.0869 | 315.0889 | 6.35 | 254.06，282.05，300.06 |
| 157 | 13.22 | ESI- | 20(s) Ginsenoside Rg2* | C_42_H_72_O_13_ | 783.4895 | 783.4877 | -2.30 | 637.44，475.37 |
| 158 | 13.22 | ESI+ | Ginsenoside Rh1* | C_36_H_62_O_9_ | 639.4472 | 639.45 | 4.38 | 393.35，163.05 |
| 159 | 13.22 | ESI- | Ginsenoside Rh8* | C_36_H_60_O_9_ | 635.4159 | 635.4169 | 1.57 | - |
| 160 | 13.22 | ESI+ | Ginsenoside Ro* | C_48_H_76_O_19_ | 957.5059 | 957.5059 | 0.00 | 957.50，439.36，339.09，204.18 |
| 161 | 13.27 | ESI+ | malonyl-ginsenoside Rb2 | C_56_H_92_O_25_ | 1165.6006 | 1165.6014 | 0.69 | 1079.05，871.02，411.51 |
| 162 | 13.4 | ESI+ | Ginsenoside Rb2* | C_53_H_90_O_22_ | 1079.6002 | 1079.601 | 0.74 | 785.50，623.39 |
| 163 | 13.4 | ESI+ | notoginsenoside Fe | C_47_H_80_O_17_ | 917.5475 | 917.5505 | 3.27 | 899.28，785.21 |
| 164 | 13.45 | ESI+ | Ginsenoside Rb3* | C_53_H_90_O_22_ | 1079.6002 | 1079.6031 | 2.69 | 785.51，623.39 |
| 165 | 13.45 | ESI+ | malonyl-ginsenoside Rc | C_56_H_92_O_25_ | 1165.6006 | 1165.6016 | 0.86 | 1015.29，853.50，835.49 |
| 166 | 13.53 | ESI+ | Ginsenoside Rh7* | C_36_H_60_O_9_ | 637.4316 | 637.43 | -2.51 | - |
| 167 | 13.58 | ESI- | Pseudoginsenoside RT1* | C_47_H_74_O_18_ | 925.4797 | 925.4777 | -2.16 | 763.01，613.01，569.00 |
| 168 | 13.58 | ESI+ | Quinquenoside R1 | C_56_H_94_O_24_ | 1151.6214 | 1151.6272 | 5.04 | 1109.30，785.21 |
| 169 | 13.66 | ESI- | apigenin | C_15_H_10_O_5_ | 269.045 | 269.0459 | 3.35 | 151.00，117.04 |
| 170 | 13.71 | ESI+ | malonyl-ginsenoside Rb3 | C_56_H_92_O_25_ | 1165.6006 | 1165.6006 | 0.00 | 1079.05，871.02，411.51 |
| 171 | 13.76 | ESI- | Rhein* | C_15_H_8_O_6_ | 283.0243 | 283.0252 | 3.18 | 239.03，211.04，183.01 |
| 172 | 13.76 | ESI+ | 20(s) Ginsenoside F1* | C_36_H_62_O_9_ | 661.4292 | 661.427 | -3.33 | 477.38 |
| 173 | 13.88 | ESI- | malonyl-ginsenoside Re | C_51_H_84_O_21_ | 1031.5427 | 1031.5399 | -2.71 | 853.00，767.02 |
| 174 | 13.89 | ESI+ | Ginsenoside Rd* | C_48_H_82_O_18_ | 947.5579 | 947.5568 | -1.16 | 785.52，623.45，461.39 |
| 175 | 13.97 | ESI+ | malonyl-ginsenoside Rd | C_51_H_84_O_21_ | 1033.5584 | 1033.5571 | -1.26 | 947.28，871.25，785.22 |
| 176 | 14.2 | ESI- | Chrysoobtusin* | C_19_H_18_O_7_ | 357.0974 | 357.099 | 4.48 | 326.99，311.80 |
| 177 | 14.32 | ESI+ | Biochanin A* | C_16_H_12_O_5_ | 285.0763 | 285.0753 | -3.51 | 267.04 |
| 178 | 14.58 | ESI+ | Ginsenoside Mb* | C_47_H_80_O_17_ | 917.5474 | 917.5503 | 3.16 | 785.50，623.45 |
| 179 | 14.76 | ESI- | Obtusin* | C_18_H_16_O_7_ | 343.0818 | 343.0816 | -0.58 | 328.06，313.04，285.04 |
| 180 | 14.76 | ESI+ | Ginsenoside Rd2* | C_47_H_80_O_17_ | 939.5293 | 939.5326 | 3.51 | 785.50，623.45 |
| 181 | 14.89 | ESI- | Tangeratin | C_20_H_20_O_7_ | 371.1131 | 371.1129 | -0.54 | - |
| 182 | 14.94 | ESI- | Obtusifolin* | C_16_H_12_O_5_ | 283.0606 | 283.0605 | -0.35 | 240.04，211.04 |
| 183 | 15.25 | ESI+ | Ginsenoside Rg6* | C_42_H_70_O_12_ | 789.4765 | 789.4814 | 6.21 | 459.38 |
| 184 | 15.43 | ESI+ | luteone* | C_20_H_18_O_6_ | 353.1082 | 353.1064 | -5.10 | 285.11， 243.10 |
| 185 | 15.43 | ESI+ | 20(s) Ginsenoside F2* | C_42_H_72_O_13_ | 785.5051 | 785.4997 | -6.87 | 461.31，163.15 |
| 186 | 15.43 | ESI+ | Ginsenoside Rg3* | C_42_H_72_O_13_ | 807.4871 | 807.4857 | -1.73 | 461.39，377.08，223.07 |
| 187 | 15.51 | ESI+ | 4',7-Dimethoxyisoflavone* | C_17_H_14_O_4_ | 283.097 | 283.099 | 7.06 | - |
| 188 | 15.56 | ESI+ | Ginsenoside Rk3* | C_36_H_60_O_8_ | 643.4186 | 643.4166 | -3.11 | 459.38，163.15 |
| 189 | 15.74 | ESI- | Emodin* | C_15_H_10_O_5_ | 269.045 | 269.0444 | -2.23 | 182.03，225.05，241.04 |
| 190 | 15.74 | ESI+ | Corylin* | C_20_H_16_O_4_ | 321.1127 | 321.114 | 4.05 | - |
| 191 | 15.74 | ESI+ | Ginsenoside Rh4* | C_36_H_60_O_8_ | 643.4186 | 643.4166 | -3.11 | 459.38，163.14 |
| 192 | 16.13 | ESI- | 20(s) Ginsenoside Rs3 | C_44_H_74_O_14_ | 825.5 | 825.5009 | 1.09 | 621.43，375.29 |
| 193 | 16.18 | ESI+ | Ginsenoside Rk1 | C_42_H_70_O_12_ | 767.4946 | 767.4952 | 0.78 | 605.44，443.38，163.06 |
| 194 | 16.43 | ESI- | Protopanaxtriol* | C_30_H_52_O_4_ | 521.3842 | 521.3829 | -2.49 | 457.99，421.98 |
| 195 | 16.48 | ESI+ | Soyasapogenol B* | C_30_H_50_O_3_ | 459.3838 | 459.3813 | -5.44 | - |
| 196 | 16.62 | ESI+ | Pseudoginsenoside Rh2* | C_36_H_62_O_8_ | 623.4523 | 623.4515 | -1.28 | - |
| 197 | 16.62 | ESI+ | Ginsenoside CK* | C_36_H_62_O_8_ | 623.4523 | 623.4515 | -1.28 | - |
| 198 | 16.74 | ESI+ | Soyasapogenol A* | C_30_H_50_O_4_ | 475.3787 | 475.3761 | -5.47 | - |
| 199 | 16.74 | ESI+ | 3'-methoxy-5'-hydroxyisoflavone-7-O-β-D-glucoside* | C_15_H_12_O_6_ | 289.0712 | 289.0729 | 5.88 | 274.05，245.04，170.87 |
| 200 | 16.79 | ESI+ | Puerol A* | C_17_H_14_O_5_ | 299.0919 | 299.0928 | 3.01 | - |
| 201 | 16.92 | ESI+ | 5-Methyl-7-methoxyisoflavone* | C_17_H_14_O_3_ | 267.1021 | 267.1033 | 4.49 | - |
| 202 | 17.23 | ESI+ | Gentisuric Acid* | C_9_H_9_NO_5_ | 212.0559 | 212.057 | 5.19 | 195.09，55.05 |
| 203 | 17.85 | ESI+ | Ginsenoside Rg5* | C_42_H_70_O_12_ | 767.4946 | 767.4959 | 1.69 | 223.11，163.06 |
| 204 | 17.85 | ESI+ | Ginsenoside Rh3* | C_36_H_60_O_7_ | 605.4417 | 605.444 | 3.80 | - |
| 205 | 17.98 | ESI- | Physcion* | C_16_H_12_O_5_ | 283.0606 | 283.062 | 4.95 | 240.04，238.99 |
| 206 | 18.03 | ESI- | 20(s) Ginsenoside Rh2* | C_36_H_62_O_8_ | 621.4366 | 621.4372 | 0.97 | - |
| 207 | 18.5 | ESI+ | Palmitic Acid* | C_16_H_32_O_2_ | 257.2402 | 257.2399 | -1.17 | - |
| 208 | 18.52 | ESI+ | Diisobutyl phthalate* | C_16_H_22_O_4_ | 279.1596 | 279.1588 | -2.87 | - |
| 209 | 18.59 | ESI+ | α-Spinasterol* | C_29_H_48_O | 413.3783 | 413.3785 | 0.48 | - |
| 210 | 19.45 | ESI+ | Ginsenoside Rk2* | C_36_H_60_O_7_ | 627.4237 | 627.4196 | -6.53 | - |

* indentified with represented standards

Table S5. Identification of 21 SHTB serum prototype components.

| **Number** | **Ion mode** | **Identification component** | **R_T_（min）** | **Molecular formula** | **Theoretical extract mass (Da)** | **Mean measured mass (Da)** | **Error (ppm)** | **Fragmetation** |
| --- | --- | --- | --- | --- | --- | --- | --- | --- |
| 1 | ESI(-) | 4-Hydroxybenzoic acid | 2.41 | C_7_H_6_O_3_ | 137.0239 | 137.0244 | 3.6 | 93.03 |
| 2 | ESI(-) | Aloesin | 2.59 | C_19_H_22_O_9_ | 393.1186 | 393.1195 | 2.3 | 273.07, 245.08 |
| 3 | ESI(-) | vanillic acid | 2.95 | C_8_H_8_O_4_ | 167.0344 | 167.0372 | 16.8 | 123.04, 107.01 |
| 4 | ESI(+) | Daidzin | 3.88 | C_21_H_20_O_9_ | 417.1186 | 417.1206 | 4.8 | 227.07, 137.03 |
| 5 | ESI(-) | Ferulic acid | 4.50 | C_10_H_10_O_4_ | 193.0501 | 193.0511 | 5.2 | 178.02, 134.03 |
| 6 | ESI(+) | Aloins | 7.40 | C_21_H_22_O_9_ | 419.1342 | 419.1302 | -9.5 | 147.03 |
| 7 | ESI(-) | Aloeresin D | 7.58 | C_29_H_32_O_11_ | 555.1866 | 555.1899 | 5.9 | 511.16, 417.12 |
| 8 | ESI(+) | Daidzein | 7.89 | C_15_H_10_O_4_ | 255.0657 | 255.0657 | 0.0 | 199.08 |
| 9 | ESI(+) | Ononin | 8.02 | C_22_H_22_O_9_ | 431.1342 | 431.1378 | 8.4 | 269.08, 163.06 |
| 10 | ESI(+) | Aloin A | 8.10 | C_21_H_22_O_9_ | 417.1186 | 417.1171 | -3.6 | 297.08, 268.07 |
| 11 | ESI(+) | Quercetin | 8.82 | C_15_H_10_O_7_ | 303.0505 | 303.0526 | 6.9 | 184.07, 109.03 |
| 12 | ESI(-) | Chrysophanol | 10.93 | C_15_H_10_O_4_ | 253.0501 | 253.0499 | -0.8 | 225.05, 181.88 |
| 13 | ESI(+) | Formononetin | 12.83 | C_16_H_12_O_4_ | 269.0814 | 269.0798 | -5.9 | 239.07 |
| 14 | ESI(-) | Ginsenoside Rb1 | 12.96 | C_54_H_92_O_23_ | 1153.6006 | 1153.5996 | -0.9 | 945.54,783.49,621.44 |
| 15 | ESI(-) | Ginsenoside Rc | 13.22 | C_53_H_90_O_22_ | 1123.59 | 1123.5908 | 0.7 | 945.54,783.49,621.44 |
| 16 | ESI(+) | GinsenosideRb2/Rb3 | 13.40 | C_53_H_90_O_22_ | 1123.59 | 1123.5902 | 0.2 | 945.54,783.49,621.44 |
| 17 | ESI(-) | Aloe-emodin | 13.40 | C_15_H_10_O_5_ | 269.045 | 269.0491 | 15.2 | 116.92, 107.05 |
| 18 | ESI(-) | Ginsenoside Rd | 13.89 | C_48_H_82_O_18_ | 991.5478 | 991.5428 | -5.0 | 783.49,621.44,459.38 |
| 19 | ESI(-) | Rhein | 13.97 | C_15_H_8_O_6_ | 283.0243 | 283.0233 | -3.5 | 239.03, 211.04,183.01 |
| 20 | ESI(-) | Obtusin | 14.95 | C_18_H_16_O_7_ | 343.0818 | 343.0818 | 0.0 | 328.06, 313.04,285.04 |
| 21 | ESI(+) | Gentisuric Acid | 17.24 | C_9_H_9_NO_5_ | 212.0559 | 212.0544 | -7.1 | 166.06, 55.05 |
| 22 | ESI(-) | Ginsenoside CK | 17.60 | C_36_H_62_O_8_ | 667.4422 | 667.4456 | 5.1 | 459.38,161.04 |

Table S6. Effect of SHTB on the fecal parameters in rats

| Group | n | The first black stool time (min) | 5h water content of rat fecal (%) | 12h water content of rat fecal (%) |
| --- | --- | --- | --- | --- |
| Control | 14 | 322.50±63.15 | 55.73±4.74 | 48.29±10.00 |
| Model | 12 | 578.333±120.70#### | 42.94±8.31### | 39.58±11.49# |
| SHTB | 13 | 328.92±59.74**** | 64.63±8.11**** | 54.52±7.71** |
| MRW | 12 | 442.42±132.67#* | 58.14±8.32**** | 51.37±10.98* |

* compared to the control group; # compared to the QS group. *p < 0.05; **p < 0.01; ****p < 0.0001. #p < 0.05; ###p < 0.001; ####p < 0.0001.

Table S7. Repeatability of the 11 internal standards for targeted lipids and fatty acids in serum

| R_T_(min) | Compound | RSD%(ESI+) | RSD%(ESI-) |
| --- | --- | --- | --- |
| 4.19 | Lyso PC19:0 | 10.43 | - |
| 4.52 | Hexadecanoic -16,16,16-D3 Acid | - | 15.46 |
| 5.15 | Stearic acid-18,18,18-d3 | - | 17.49 |
| 5.46 | PG(14:0/14:0) | - | 15.26 |
| 5.65 | Nonadecanoic acid | - | 16.46 |
| 5.86 | PI(17:0/14:0) | - | 21.24 |
| 6.18 | PE(12:0/13:0) | 10.2 | - |
| 6.4 | SM(d18:0/12:0) | 9.22 | - |
| 9.49 | Cer(d18:1/17:0) | 8.96 | - |
| 10.95 | PC(19:0/19:0) | 10.29 | - |
| 13.17 | TG (15:0/15:0/15:0) | 9.74 | - |

Table S8. Repeatability of the internal standards for SCFA analyses in serum and feces

| R_T_(min) | Compound | RSD%(Serum) | RSD%(Feces) |
| --- | --- | --- | --- |
| 6.9 | 2-Methylvaleric acid | 10.59 | 10.6 |

1. Guo, N.; Wang, P.; Yang, J.; Yang, X.; van der Voet, M.; Wildwater, M.; Wei, J.; Tang, X.; Wang, M.; Yang, H. Serum Metabolomic Analysis of Coronary Heart Disease Patients with Stable Angina Pectoris Subtyped by Traditional Chinese Medicine Diagnostics Reveals Biomarkers Relevant to Personalized Treatments. *Front. Pharmacol.* **2021**, *12*, 664320, doi:10.3389/fphar.2021.664320.
